# Species-Specific Immunological Reactivities Depend on the Cell-Wall Organization of the Two *Aspergillus*, *Aspergillus fumigatus* and *A. flavus*

**DOI:** 10.3389/fcimb.2021.643312

**Published:** 2021-02-25

**Authors:** Sarah Sze Wah Wong, Lakshmi Prabha Venugopalan, Audrey Beaussart, Anupama Karnam, Mohammed Razeeth Shait Mohammed, Jeya Maheshwari Jayapal, Stéphane Bretagne, Jagadeesh Bayry, Lalitha Prajna, Dharmalingam Kuppamuthu, Jean-Paul Latgé, Vishukumar Aimanianda

**Affiliations:** ^1^ Institut Pasteur, Unité des *Aspergillus*, Paris, France; ^2^ Institut Pasteur, Molecular Mycology Unit, CNRS, UMR-2000, Paris, France; ^3^ Department of Proteomics & Ocular Microbiology, Aravind Medical Research Foundation, Madurai, India; ^4^ Université de Lorraine, CNRS, LIEC, Nancy, France; ^5^ Institut National de la Santé et de la Recherche Médicale, Centre de Recherché des Cordeliers, Sorbonne Université, Université de Paris, Paris, France; ^6^ Department of Ocular Microbiology, Aravind Eye Hospital, Madurai, India

**Keywords:** *Aspergillus*, conidia, cell-wall organization, polysaccharides, immunoreactivity, antifungal, drug susceptibility

## Abstract

Although belong to the same genus, *Aspergillus fumigatus* is primarily involved in invasive pulmonary infection, whereas *Aspergillus flavus* is a common cause of superficial infection. In this study, we compared conidia (the infective propagules) of these two *Aspergillus* species. In immunocompetent mice, intranasal inoculation with conidia of *A. flavus* resulted in significantly higher inflammatory responses in the lungs compared to mice inoculated with *A. fumigatus* conidia. *In vitro* assays revealed that the dormant conidia of *A. flavus*, unlike *A. fumigatus* dormant conidia, are immunostimulatory. The conidial surface of *A. fumigatus* was covered by a rodlet-layer, while that of *A. flavus* were presented with exposed polysaccharides. *A. flavus* harbored significantly higher number of proteins in its conidial cell wall compared to *A. fumigatus* conidia. Notably, β-1,3-glucan in the *A. flavus* conidial cell-wall showed significantly higher percentage of branching compared to that of *A. fumigatus*. The polysaccharides ensemble of *A. flavus* conidial cell wall stimulated the secretion of proinflammatory cytokines, and conidial cell wall associated proteins specifically stimulated IL-8 secretion from the host immune cells. Furthermore, the two species exhibited different sensitivities to antifungal drugs targeting cell wall polysaccharides, proposing the efficacy of species-specific treatment strategies. Overall, the species-specific organization of the conidial cell wall could be important in establishing infection by the two *Aspergillus* species.

## Introduction

Globally, chronic, allergic, and invasive forms of fungal infections account for over 1.6-million deaths annually; *Aspergillus* species are among the leading cause for mortality (Denning and Bromley, 2015; Bongomin et al., 2017; Cole et al., 2017). The genus *Aspergillus* consists of a few hundred species (Geiser, 2009); however, the most common pathogenic species are *A. fumigatus*, *A. flavus*, *A. terreus*, and *A. niger* (Oliveira et al., 2015). These species are saprophytes with ubiquitous distribution, and are responsible for a wide clinical spectrum (Tekaia and Latge, 2005; Amaike and Keller, 2011). *A. fumigatus* is by far the main etiologic agent causing invasive pulmonary aspergillosis (IPA) in immunocompromised hosts, and also the most common species involved in allergic broncho-pulmonary aspergillosis (ABPA) and chronic pulmonary aspergillosis (CPA) (Dogra et al., 2016). *A. flavus* is one of the leading causes of fungal keratitis, a superficial infection of the cornea (Leema et al., 2010; Ansari et al., 2013; Al-Hatmi et al., 2019). Nevertheless, *A. flavus* is also the predominant species causing IPA in Africa, Asia and Middle East, which is attributed to its ability to survive in humid environment (Rudramurthy et al., 2019).

*Aspergillus* species produces airborne conidia that serve as the infective propagule through inhalation. The aim of our study was to examine differences in the conidia of *A. fumigatus* and *A. flavus* that could explain their clinical patterns. We focused on conidial surfaces, representing the first component to interact with the host immune system. Our results demonstrate that in contrast to *A. fumigatus*, *A. flavus* conidia were heterogeneous in size and surface organization. *A. flavus* conidia showed cell surface exposed polysaccharides in contrast to *A. fumigatus* conidia, which resulted in differential immuno-stimulatory properties of these two *Aspergillus* species. These species-specific characteristics could drive their corresponding efficiency in entry and survival in the host, contributing to the observed preferred sites of infections.

## Materials and Methods

### Fungal Strains, Media, and Reagents

A clinical isolate of *A. fumigatus*, CBS144-89, from an invasive aspergillosis patient (Thau et al., 1994) and a corneal isolate of *A. flavus*, CI1698, were the strains used in this study. Other clinical isolates (twelve each for *A. fumigatus* and *A. flavus*) were obtained from the National Reference Center for Invasive Mycoses & Antifungals in Institut Pasteur. These clinical isolates were isolated from the eye/cornea, sinus and bronchoalveolar lavage fluid (**Supplementary Table 2**). All the isolates were maintained on 2% malt agar slants at ambient temperature. Conidia were harvested from 12–15-days old slants using 0.05% Tween-water, washed twice, re-suspended in Tween-water and filtered through 40-µm Falcon™ cell strainer (Thermo Fisher Scientific) to remove any mycelia. Germinating conidia were prepared by incubating dormant conidia in Sabouraud liquid medium in an incubator maintained at 150 rpm and 37°C for 6–6.5 h, followed by collecting and washing them with water. Dormant conidial inactivation by *para-*formaldehyde (PFA) was performed as described earlier (Aimanianda et al., 2009).

### Staining and Immunolabeling

Conidia were stained with Calcofluor White (CFW, 5 µg/ml) and observed under fluorescent microscope (EVOS FL Cell Imaging System, Life Technologies, Thermo Fisher Scientific). For Concanavalin-A (ConA) and wheat germ agglutinin (WGA) staining, conidia were washed extensively with PBS containing 1% BSA (PBS-BSA) and incubated for 30 min in dark with ConA/WGA conjugated with fluorescein isothiocyanate (FITC) at a concentration of 5 µg/ml constituted in PBS-BSA. Following, conidia were washed three times with PBS and observed under fluorescent microscopy (EVOS FL Cell Imaging System) (Alsteens et al., 2013). β-1,3-Glucan and α-1,3-glucan labeling for dormant conidia, and GAG labeling for germinating conidia were performed following the protocols described earlier (Aimanianda et al., 2009; Fontaine et al., 2011; Valsecchi et al., 2020). Permeabilization of the germinating conidia was performed similar to dormant conidia (Mouyna et al., 2016) but prolonging the incubation with Glucanex (Novozymes) for 60 min. All the fluorescent labeling was performed with PFA-fixed dormant or germinating conidia.

### Conidiation, Radial Growth and Germination

Conidial suspensions (100 µl, 10^4^ conidia/ml) were inoculated into three malt agar slants (2%). After 3–15 days at ambient temperature, conidia were recovered with 2 ml of 0.05% Tween-water and counted (Jimenez-Ortigosa et al., 2012). Radial growths were measured on Sabouraud-agar medium in petri-plates upon spotting 5000 conidia and incubating for 48 h at 37°C. Radial growths at different temperatures (ambient, 30°C, 37°C and 45°C) were checked on 2% malt-agar spread over petri-plates. Germination rates were investigated by inoculating dormant conidia in Sabouraud liquid medium (10^5^ conidia/ml, 6 h at 37°C), followed by observing them under microscope (EVOS FL Cell Imaging System). Resazurin growth assay was performed as described elsewhere (Clavaud et al., 2012).

### Atomic Force Microscopy (AFM)

Performed in deionized water using a Fastscan Dimension Icon (Bruker Corporation, Santa Barbara, CA). Images were recorded in peak force tapping mode using oxide sharpened micro-fabricated Si_3_N_4_ coated with Au cantilevers with a nominal spring constant of ~0.24 N/m (Bruker Corporation). Conidia were harvested from 12–15 days old malt agar slants maintained at ambient temperature, washed extensively with Tween-water, followed by washing twice with MilliQ water, subjected to PFA-fixation (Aimanianda et al., 2009) and then immobilized by mechanical trapping into porous polycarbonate membranes (Millipore). After filtering a concentrated conidial suspension, the filter was gently rinsed with deionized water, carefully cut, attached to a steel sample puck using a small piece of double face adhesive tape, and the mounted sample was transferred into the AFM liquid cell while avoiding dewetting. Data processing was performed using the commercial Nanoscope Analysis software (Bruker Corporation). At least eight conidia were analyzed to determine the percentage of conidia completely and partially covered by the surface rodlet-layer.

### Mass Spectrometric Analysis of Conidial Surface Proteins

Formic acid (95%) was added to conidia and incubated at 4°C (*A. fumigatus* conidia for 2.5 h and *A. flavus* conidia for 1 h). Upon centrifugation, the supernatants were separated, dried under nitrogen, co-evaporated twice with water and dissolved in water. Further, these FA extracts were resolved on an 8-16% gradient gel. The Coomassie Brilliant Blue stained gel was subjected to densitometry analysis and the concentration of the two RodA isoforms was determined as band density. Since both protein bands were identified as RodA, the sum of the band densities for B1 and B2 was considered 100%, and the percentages of B1 and B2 was calculated accordingly.

Formic acid extracts were also subjected to in-solution tryptic digestion followed by nanoLC-orbitrap mass spectrometry analysis. All MS/MS raw data acquired from Orbitrap Velos Pro mass spectrometer were analyzed by Proteome Discoverer v1.4 using Mascot (Matrix Science, London, UK; version 2.4.1.0) as well as with an inbuilt SequestHT algorithm. Both Mascot and SequestHT was set up to search a database containing the complete proteome of *A. flavus* NRRL3357 (13,500 entries) or *A. fumigatus Af*293 (9648 entries) downloaded from the UniProt database on September 2018. The database search was performed with the following parameters: peptide tolerance of 10 ppm and fragment tolerance of 0.60 to 0.80 Da, allowing two missed cleavages with trypsin as the enzyme. Cysteine carbamidomethylation was given as fixed modification while methionine oxidation, *N*-terminal acetylation and phosphorylation (S, T, Y) as variable modifications. The peptide spectrum matches (PSMs) from SequestHT and Mascot were post-processed using the Percolator algorithm (Kandhavelu et al., 2017). Those peptides having a q-value lesser than the threshold of 0.01 were considered for protein identification. Proteins with minimum three PSMs were considered. The cellular localization of the proteins was referred to by the Gene ontology (cellular component) entry on UniProt, and the proteins were classified as cell surface proteins and/or intracellular proteins.

### Conidial/Mycelial Cell Wall Analysis

Dormant conidia harvested from malt-agar slants after 12–15 days growth at ambient temperature or mycelia collected by filtration after 24 h of growth in Sabouraud liquid medium maintained at 37°C in a shaking incubator were subjected to cell wall analysis as described earlier (Jimenez-Ortigosa et al., 2012).

### Isolation and Stimulation Of Immune Cells, and Cytokine Analysis

*(i) Human monocyte-derived macrophages:* Blood samples from healthy donors were obtained from Etablissement Français du Sang (Saint-Louis hospital, Paris, France) with written and informed consent as per the guidelines provided by the institutional ethics committee, Institut Pasteur (convention 12/EFS/023). The peripheral blood mononuclear cells (PBMCs) were isolated by a density-gradient separation using ficoll (Eurobio, France). Isolated PBMCs were re-suspended in RPMI 1640 medium (Gibco) and seeded in each well (500 µl containing 2x10^6^ PBMCs) of 12-well microtiter plates (Nunc Labware products; Sigma-Aldrich). After overnight incubation, the cells were washed twice with PBS. Then, RPMI medium supplemented with 10% normal human serum (NHS) and granulocyte macrophage colony stimulating factor (GM-CSF; 10 ng/ml; Sigma-Aldrich) was added for monocyte differentiation into macrophage (MDMs). After 6 days, medium was discarded; differentiated MDMs were washed with PBS and used for further experiments. *(ii) Neutrophils* – Isolated from whole blood using EasySep™ direct human isolation kit (Stemcell Technologies) and seeded (1x10^6^/well) into 12-well microtiter plates. *(iii) Dendritic cells (DCs) –* Generated using PBMCs isolated from whole blood samples of healthy human donors obtained from the Centre Necker-Cabanel, Etablissement Français du Sang (EFS), Paris (Maddur et al., 2014). Institut National de la Santé et de la Recherche-EFS ethical committee permission (N°18/EFS/033) has been obtained for the use of human samples.

MDMs (obtained after seeding 2x10^6^ PBMCs per well), DCs or neutrophils (5x10^5^/well) were incubated with 5x10^5^ conidia per well in RPMI for 20 h at 37°C in a CO_2_ incubator. The co-culture supernatants were collected and stored at -20°C until further analysis. Cytokines (TNF-α, IFN-γ IL-1β, IL-2, IL-6, IL-8, IL-10, IL-12p70) in the supernatants were quantified using DuoSet ELISA kits (R&D Systems). MDMs were also incubated with conidial cell wall alkali-insoluble (AI), soluble (AS) fractions (10 μg/well) and formic acid extracts (at protein concentrations of 1 μg/well) for 20 h at 37°C in a CO_2_ incubator. The co-culture supernatants were analyzed for cytokines using DuoSet ELISA kits.

### Murine Model to Study the Immunostimulatory Capacities of the Two *Aspergillus* Species

The animal experiments were approved by the ethical committee for animal experimentation Comité d’Éthique en Experimentation Animale (CETEA Project license number 2013-0055). Three groups of mice, each containing three mice, were inoculated intranasally with 25 µl PBS (control) or 1x10^7^ conidia of *A. fumigatus* or *A. flavus* suspended in 25 µl of PBS (test). After 6 h, mice were sacrificed, their lungs were collected, homogenized with PBS (pH 7.4) supplemented with 0.5% Triton X-100, 0.02% sodium azide, phenylmethylsulfonyl fluoride (PMSF; 1 mM), and protease inhibitor cocktail (Roche cOmplete™ EDTA-free protease inhibitor cocktail tablet), and the clear supernatant collected as described earlier (Wong et al., 2018). Further, protein concentration equivalent to 20 µg (as determined by Bradford assay using BSA standard) was taken for cytokine estimation using DuoSet ELISA kits (R&D Systems).

Male BALB/c mice (8-weeks old, 5-mice per group in each set of experiment, performed twice) were immunosuppressed by intraperitoneal injections of cyclophosphamide (200 mg/kg) on day -4 and day -1. Prior to the infection on day 0, each mouse was anesthetized by intramuscular injection of 10 mg/ml ketamine and 10 mg/ml xylazine (150 μl). Following, each mouse was intranasally challenged with 5x10^5^ or 5x10^4^ conidia suspended in 25 μl PBS with 0.05% Tween (PBS-Tween) and their survival was monitored; the control mice received 25 μl PBS-Tween.

### Susceptibility of the Two *Aspergillus* Species to Cell Wall Perturbing Agents and Antifungals

*(i) Cell wall perturbing agents:* Congo red (CR) and Calcofluor white (CFW) were added to malt-agar medium (1 ml/well into a 6-well plate, at 0–250 µg/ml concentrations). Conidia (5,000/well) were seeded on this agar medium and incubated at 37°C for 24 h. Following, the radial growths were measured. For Menadione (starting at 160 µM with two-fold dilutions) and H_2_O_2_ (starting at 6 µM with two-fold dilutions) assays, resazurin method was followed (Clavaud et al., 2012). *(ii) Antifungal drug susceptibility assay:* Performed by resazurin method as described earlier (Clavaud et al., 2012). Briefly, yeast-extract medium (1%; 9 ml of the medium containing 9 μl Tween-20) was mixed with 3 ml of resazurin (1 mg/ml dissolved in Milli-Q water) and 1 ml of conidial suspension (containing 1x10^4^ conidia/ml); 130 μl of this mixture was seeded into each well in 96-well culture plate. Two-fold dilution of caspofungin (starting at 0.72 μg/well) or nikkomycin (starting at 1.92 μg/well) were added to these well, the culture-plates were incubated at 37°C for 30 h and the optical densities were measure at 569 nm using Tecan plate-reader Infinite 200 PRO (Tecan Group Ltc., Switzerland).

### Statistical Analysis

Performed using Prism-8 software (GraphPad Software, Inc., La Jolla, CA, USA), analyzing the data by one-way repeated measure (RM) ANOVA, two-way ANOVA with Tukey’s multiple comparison test or nonparametric Mann–Whitney test.

## Results

### Immunogenic Potentials of the Two *Aspergillus* Species Are Different

**Figure 1:**
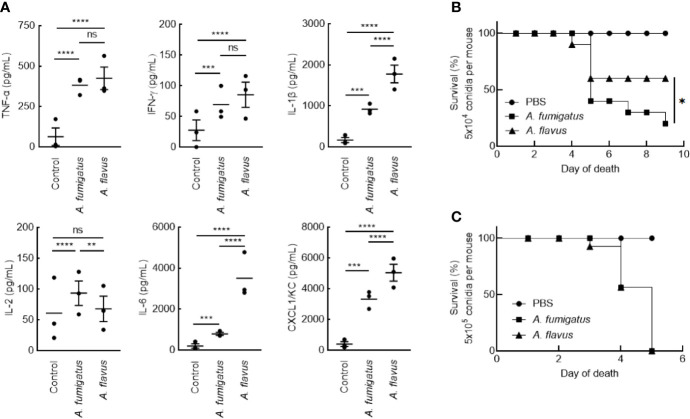
**(A)** Lung-cytokine analyses upon infecting mice with *Aspergillus* conidia. Immunocompetent mice were infected intranasally with *A. fumigatus* or *A. flavus* conidia (1x10^7^/mouse; control mice were injected with PBS). Mice were sacrificed after 6 h, their lungs were collected, homogenized and the cytokines in the supernatant were estimated by ELISA (Mean ± standard deviation; ns – not significant, **p < 0.05, ***p < 0.005 and ****p < 0.0005). **(B, C)** Survival of mice infected with conidia. BALB/c mice were immunosuppressed by intraperitoneal injections of cyclophosphamide (200 mg/kg) on day -4 and day -1. On day 0, prior to the infection, each mouse was anesthetized by intramuscular injection of 10 mg/mL ketamine and 10 mg/ml xylazine (150 μl). Following, each mouse was intranasally inoculated with **(B)** 5x10^4^ or **(C)** 5x10^5^ conidia; control mice received 25 μl PBS-Tween. Upon 5x10^4^ conidial inoculation, *A. fumigatus* infected mice showed significantly lower survival rate (20%) compared to *A. flavus*-infected mice (60%) (*p < 0.05).

Clinical isolates of *A. fumigatus* (CBS144-89) and *A. flavus* (CI1698) were used for this study. In immunocompetent mice, intranasal inoculation of the conidia of *A. fumigatus* or *A. flavus* (1x10^7^ per mouse), both stimulated inflammatory immune responses in the mice lungs. However, *A. flavus* conidia stimulated significantly higher amounts of IL-6, IL-1β, CXCL1/KC, and lower level IL-2 secretions in the lungs (**Figure 1**), suggesting that *A. flavus* conidia trigger higher proinflammatory immune response as compared to *A. fumigatus* conidia. There was no difference in the IL-10 level in the lungs of the mice inoculated with either of the *Aspergillus* conidia (in pg/ml: *A. fumigatus* - 611.94 ± 160.72, *A. flavus* - 616.55 ± 232.22, and control - 213.33 ± 174.88).

In immunosuppressed mice, intranasal inoculation of 5x10^4^ conidia per mouse resulted in a significant difference in the survival of the mice infected with *A. fumigatus* and *A. flavus* conidia (**Figure 1B**; 20% and 60% for *A. fumigatus* and *A. flavus*, respectively). However, at a higher inoculum size of 5x10^5^ conidia per mouse, both *A. fumigatus* and *A. flavus* were equally virulent and showed comparable survival (**Figure 1C**), suggesting that the conidial number has an impact on the virulence of *A. flavus.*

**Figure 2:**
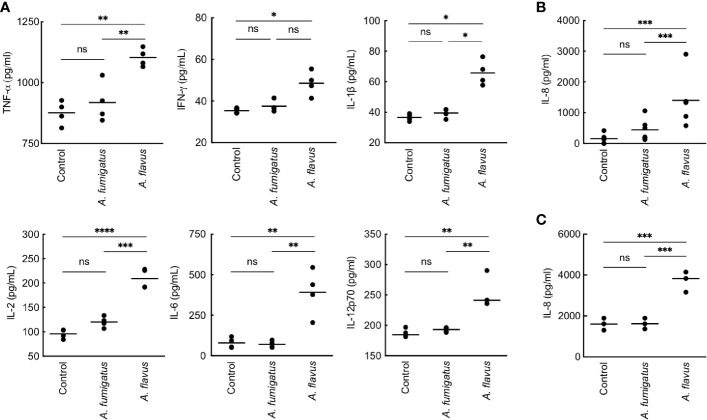
Cytokine induction in human monocyte-derived macrophages (MDMs) by *para-*formaldehyde (PFA)-fixed (inactivated) conidia. **(A)** Human MDMs [obtained by seeding 2x10^6^ peripheral blood mononuclear cells (PBMCs) per well] of four healthy donors were co-incubated with PFA-fixed conidia (5x10^5^ per well) in complete RPMI (37°C for 20 h). The supernatants were collected for cytokine quantification (TNF-α, IFN-γ, IL-1β, IL-2, IL-6, and IL-12p70). *A. fumigatus* conidia did not induce cytokine production from MDMs. However, *A. flavus* conidia significantly stimulated the secretion of pro-inflammatory cytokines TNF-α, IL-1β, IL-2, IL-6, IL-12p70, and IL-8 (*p < 0.05, **p < 0.01, ***p < 0.005, ****p < 0.0005). While *A. flavus* conidia specifically stimulated the secretion of IL-8 by dendritic cells **(B)** and neutrophils **(C)** (***p < 0.005). ns – not significant.

**Figure 3:**
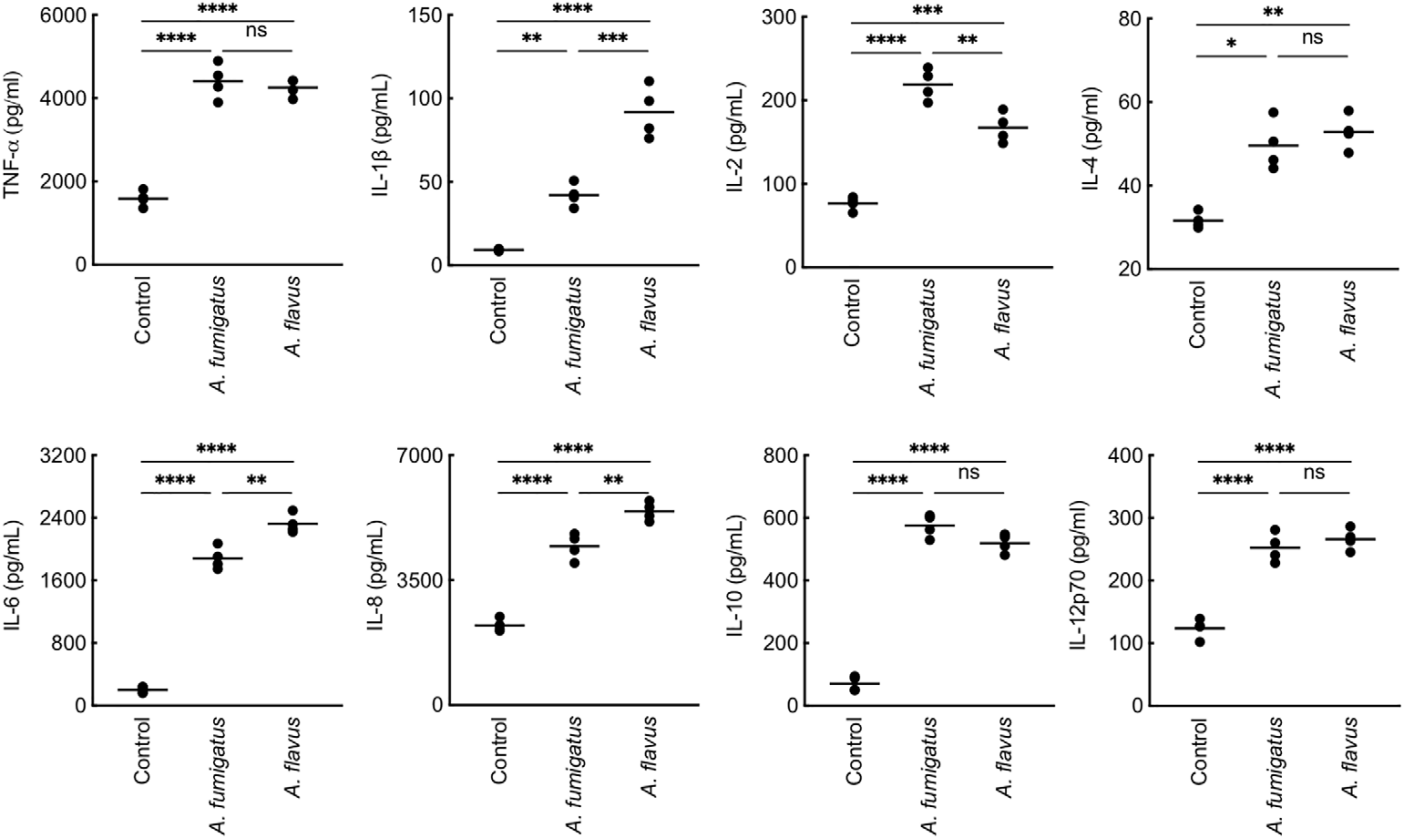
Cytokine induction in human monocyte-derived macrophages (MDMs) by metabolically active conidia. Human MDMs [obtained by seeding 2x10^6^ peripheral blood mononuclear cells (PBMCs) per well] of four healthy donors were co-incubated with metabolically active (live) conidia (5x10^5^ per well) in RPMI (37°C, 20 h), and the supernatants were collected for cytokine quantification. Both *A. fumigatus* and *A. flavus* stimulated the cytokine secretion by MDMs. *A. fumigatus* live conidia stimulated a significantly higher level of IL-2 compared to *A. flavus*, while *A. flavus* stimulated higher secretion of IL-1β and IL-6 (ns – not significant, *p < 0.05, **p < 0.01, ***p < 0.005 and ****p < 0.0005).

We then studied conidial interaction with human monocyte derived macrophages (MDMs), neutrophils and dendritic cells (DCs), the major phagocytes and/or antigen presenting cells present in the lung alveoli or cornea (Akpek and Gottsch, 2003; Suzuki et al., 2008; Palomar et al., 2019). Both *p-*formaldehyde fixed (PFA; inactivated) and metabolically active (live) conidia were used for this study. The panel of cytokine tested was TNF-α, IFN-γ, IL-1β, IL-2, IL-6, IL-8, IL-10, and IL-12p70, involved in inflammation, recruitment of immune cells and T cell polarization. Inactivated conidia of *A. fumigatus* failed to stimulate the secretion of any cytokines by MDMs, neutrophils, and DCs. In the contrary, *A. flavus* conidia, even though inactivated, stimulated the secretion of cytokines TNF-α, IL-1β, IL-2, IL-6, IL-12p70 (**Figure 2A**), and IL-10 from MDMs, and specifically stimulated the secretion of IL-8 by DCs (**Figure 2B**) and neutrophils (**Figure 2C**). Metabolically active (live) conidia of both *A. fumigatus* and *A. flavus* stimulated the secretion of cytokines by MDMs and their stimulatory potentials were comparable for TNF-α, IL-4, IL-10, and IL-12p70 (**Figure 3**). Nevertheless, there was significantly higher production of IL-1β, IL-6, IL-8, and decreased secretion of IL-2 by MDMs interacted with *A. flavus* conidia compared to *A. fumigatus* conidial stimulation (**Figure 3**).

### The Two *Aspergillus* Species Showed Differential Growth on Solid and in Liquid Medium

When grown on Sabouraud agar medium, there was no difference in conidiation between the two *Aspergillus* species (**Supplementary Figure 1A**). However, *A. flavus* showed slightly lower radial growth (**Supplementary Figure 1B**; 2.4 ± 0.1 cm and 1.7 ± 0.1 cm for *A. fumigatus* and *A. flavus*, respectively, after 48 h of growth at 37°C). On the contrary, after 6 h of incubation at 37°C in the Sabouraud liquid medium, *A. flavus* conidia formed germ tubes whereas *A. fumigatus* conidia remained in the swollen state, suggesting a faster germinating capacity of *A. flavus* conidia (**Supplementary Figure 1C**). *A. flavus* was less thermotolerant compared to *A. fumigatus*: radial growth of *A. flavus* at 30°C was more than *A. fumigatus*, their growths were similar at 37°C, and at 45°C only *A. fumigatus* was able to grow (**Supplementary Figure 1D**).

### *A. flavus* Conidia Are Heterogeneous in Size and with Surface Exposed Polysaccharides

*A. flavus* conidia from the 10-day old culture slant were heterogeneous in size (5.2 ± 1.9 µm), unlike *A. fumigatus* conidia that were uniform and smaller with an average size of 2.6 ± 0.2 µm (**Supplementary Figure 2A**). We also collected conidia from 3–15 days cultures to determine whether the heterogeneous conidial sizes of *A. flavus* are associated with age. The heterogeneity in the conidial size was equally observed from 3 to 15-days, suggesting that it is an inherent characteristic of *A. flavus* conidia. *A. fumigatus* conidia were uniformly labeled with Calcofluor White (CFW; for chitin labeling), but *A. flavus* conidia showed heterogeneous labeling; at least 50% of *A. flavus* conidia were completely or partially labeled, and the rest were not labeled (**Supplementary Figure 2B**). Concanavalin A (ConA)-FITC or wheat germ agglutinin (WGA)-FITC (for surface mannan and glucosamine, respectively) labeling were also heterogeneous for *A. flavus* conidia. *A. fumigatus* conidia were not labeled by either ConA-FITC nor WGA-FITC (**Supplementary Figure 3**), suggesting the absence of mannan and glucosamine moieties on the *A. fumigatus* conidial surface.

### The Two *Aspergillus* Species Demonstrated Differential Cell Wall Proteomes

**Figure 4:**
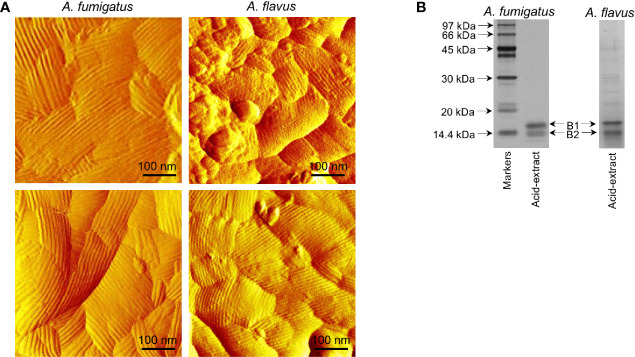
Comparison of the conidial surfaces and extractable proteins from the conidial surface of *A. fumigatus* and *A. flavus*. **(A)** Atomic force microscopy images of the conidial surfaces acquired in liquid conditions. Conidial surface of *A. fumigatus* is uniformly covered by rodlet layer, whereas amorphous material was observed along with the rodlets on *A. flavus* conidial surfaces. **(B)** SDS-PAGE profiles of the conidial formic acid-extracts showing two isoforms of RodA [B1 and B2, with experimentally determined molecular weights of 16 kDa (B1) and 14.5 kDa (B2), respectively].

Imaging by atomic force microscopy (AFM) indicated that the *A. fumigatus* conidial surfaces are uniformly covered by a rodlet layer of hydrophobic protein RodA (**Figure 4A**) (Alsteens et al., 2013; Ansari et al., 2013). *A. flavus* conidial surfaces were heterogeneous, about 40% conidia exhibited well-formed rodlet layer, the rest were presented with amorphous material besides the rodlets. The rodlets on *A. fumigatus* conidial surface were comparatively longer than those found on the *A. flavus* conidial surface.

SDS-PAGE profile of the *A. fumigatus* conidial surface protein extracted by formic acid showed mainly RodA, whereas there were additional protein bands along with RodA in the *A. flavus* conidial extract (**Figure 4B**). There were two proteoforms of RodA, corresponding to experimentally determined molecular weights of 16 kDa (B1) and 14.5 kDa (B2) (**Figure 4B**) (Aimanianda et al., 2009), in both the *Aspergillus* species. Mass spectrometry analysis of the excised bands confirmed the identities of the two proteoforms of RodA of both *Aspergillus* species. These two forms differed in their relative proportions in the two *Aspergillus* species. In *A. flavus*, the 16 kDa (B1) and 14.5 kDa (B2) forms constituted 45% and 55% of the RodA, respectively, whereas in *A. fumigatus*, B1 was more abundant (76%) than B2 (24%) of the total RodA.

Mass spectrometry analysis was also performed to identify the proteins in the conidial formic acid extracts. It should be noted that *A. flavus* conidia were subjected to formic acid extraction at 4°C for 1 h. However, under this condition, significantly less amount of protein could be extracted from *A. fumigatus* conidia. Therefore, the extraction duration was prolonged to 2.5 h for *A. fumigatus*. A total of 192 and 229 proteins were identified (with two or more identified unique peptides), respectively, in *A. flavus* and *A. fumigatus*. Among the 192 proteins in the formic acid extract from *A. flavus* conidia, 22 (11%) were cell surface proteins, 63 (33%) were intracellular proteins, and no information was available for the remaining 107 (56%). In the formic acid extract from *A. fumigatus* conidia, 51 (22%) were cell surface proteins, 104 (45%) were intracellular proteins, and the remaining 74 proteins (32%) were of unknown localization. The peptide spectrum match (PSM) score was the highest for hydrophobin in the acid extract of *A. fumigatus*. Whereas for *A. flavus*, the highest PSM score was observed for a putative GPI anchored protein, and this score was relatively lower for hydrophobin compared to *A. fumigatus* (**Supplementary Table 1A, B**). Each of these *Aspergillus* species had unique allergens in their extracts [Asp F15 in *A. fumigatus*; Asp F9 (an extracellular cell wall glucanase; Crf1), ceratoplatanin (Asp F13), enolase (Asp F22), and alkaline protease in *A. flavus*]. Other potential virulence factors found only in the formic acid extract of *A. flavus* conidia included superoxide dismutase and Hsp70.

### Cell Wall of *A. flavus* Contained More Fibrillar Polysaccharides Compared to *A. fumigatus* Cell Wall

We compared the cell wall composition of both conidial and mycelial morphotypes of the two *Aspergillus* species. From the cell wall, the core fibrillar polysaccharides could be extracted as alkali-insoluble (AI) fraction, and the amorphous polysaccharides as alkali-soluble (AS) material. In the conidial cell wall, the AI : AS ratios were 1.8 ± 0.2 and 4.1 ± 0.2 for *A. fumigatus* and *A. flavus*, respectively, suggesting the presence of more fibrillar polysaccharides in the *A. flavus* conidial cell wall. In the mycelial cell wall, the AI : AS ratio remained similar as in the conidial cell wall for *A. fumigatus* (2.0 ± 0.2). In *A. flavus*, however, this ratio was further increased to 5.0 ± 0.3, suggesting an increase in fibrillar polysaccharides in the mycelial cell wall.

**Figure 5:**
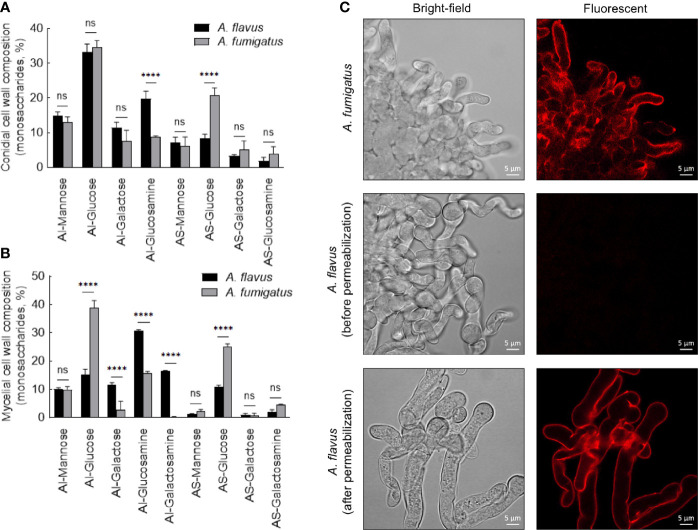
Conidial **(A)** and mycelial **(B)** cell wall composition in the two *Aspergillus* species. The alkali-insoluble (AI) fraction containing fibrillar polysaccharides and alkali-soluble (AS) fraction with amorphous component were subjected to gas-liquid chromatography after derivatization, to determine their monosaccharide compositions (three biological replicates; ****p < 0.0005, ns – not significant, error bars – standard deviation). **(C)** Localization of galactosaminogalactan (GAG). Monoclonal anti-GAG antibody was added to the germinating conidia, with or without cell wall permeabilization by Glucanex, followed by incubation with anti-mouse IgG conjugated to TRITC.

In the conidial cell wall, there were significantly higher chitin (AI-glucosamine) and lower α-1,3-glucan (represented by AS-glucose) contents in *A. flavus* compared to *A. fumigatus* (**Figure 5A**). In the mycelial cell wall, in addition to the differences in the chitin and α-1,3-glucan levels, there was a significant decrease in the β-1,3-glucan content (AI-glucose) in *A. flavus* in comparison with *A. fumigatus* (**Figure 5B**). Otherwise, the mannose and galactose contents (representing the galactomannan) in the conidial and mycelial cell wall were comparable for both the *Aspergillus* species. There were significantly higher amounts of galactose and galactosamine [representing galactosaminogalactan (GAG)] in the AI fraction of *A. flavus* mycelial cell wall. However, GAG was found in the AS fraction of *A. fumigatus* cell wall, and its amount was not significantly different compared to that in the AS fraction of *A. flavus*. Immunolabeling with monoclonal anti-GAG antibody was positive for germinating conidia of *A. fumigatus* but not of *A. flavus* (**Figure 5C**). However, a positive labeling was observed in *A. flavus* mycelia only after permeabilization of the cell wall (**Figure 5C**), confirming that GAG is an integral part of the fibrillar component (AI fraction) in the mycelial cell wall of *A. flavus*.

### Other Clinical Isolates of *Aspergillus* Display Species-Specific Characteristics

We examined the conidial size, surface labeling and growth for twelve clinical isolates each of *A. fumigatus* and *A. flavus* recovered from different sites of infections (eye, sinus, and lungs; **Supplementary Table 2**). All the twelve isolates of *A. fumigatus* were homogenous in their conidial sizes, refractory to ConA-/WGA-FITC labeling, and displayed growth at different temperatures similar to that of the *A. fumigatus* CBS144-89 isolate. The twelve isolates of *A. flavus* were similar to the CI1698 isolate for their conidial sizes, ConA-/WGA-FITC labeling, and also for their growth at different temperatures (**Supplementary Figure 4**). We then randomly selected a clinical isolate each for *A. fumigatus* and *A. flavus* along with *A. flavus* ATCC strain for their cell wall analyses. *A. fumigatus* isolate Afs35 (from an aspergilloma patient) showed mycelial cell wall composition similar to that of the CBS144-89 strain, whereas the *A. flavus* clinical isolate (A1421; from a keratitis patient) and the ATCC strain cell wall compositions were comparable to that of the *A. flavus* CI1698 isolate (**Supplementary Figure 5**). These data suggested that *A. fumigatus* and *A. flavus* show species-specific characteristics regardless of their origin.

### *A. flavus* Conidial Cell Wall Components Lead to Immunostimulation

**Figure 6:**
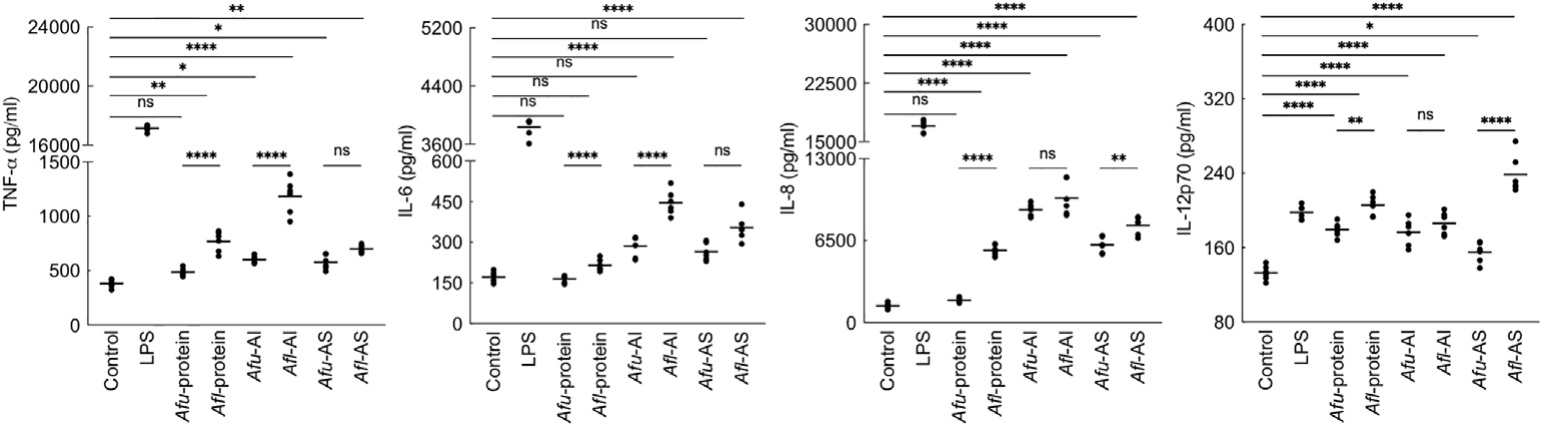
Cytokine induction by the conidial cell wall components of the two *Aspergillus* species. Human monocyte-derived macrophages obtained by seeding 2x10^6^ PMBCs per well were co-incubated with conidial cell wall alkali-insoluble (AI), soluble (AS) fractions (10 μg per well), or conidial formic acid extracts (protein; 1 μg per well) in RPMI medium at 37°C in CO_2_ incubator for 20 h. Following, the supernatants were collected for cytokine quantification (macrophages obtained from three heathy donors and two technical replicate-data are presented; *p < 0.05, **p < 0.01, ***p < 0.005 and ***p < 0.0005; ns – not significant, bar – median, Afu: *A. fumigatus* and Afl: *A. flavus*).

As the PFA-fixed *A. flavus* conidia elicited immune response (**Figure 2**), we investigated the immunomodulatory capacity of its conidial cell wall components. MDMs were stimulated with the proteins and polysaccharide ensembles extracted from the conidia of both *Aspergillus* species (**Figure 6**). *A. fumigatus* conidial protein extract stimulated the secretion of IL-12p70 only, while there was significantly higher secretion of TNF-α, IL-6, IL-8, and IL-12p70 by MDMs stimulated with *A. flavus* conidial protein extract. Both AI (composed of β-1,3-glucan, galactomannan, and chitin) and AS (composed of α-1,3-glucan and galactomannan) fractions of the conidial cell wall of *A. fumigatus* and *A. flavus* could stimulate the secretion of TNF-α, IL-6, IL-8, and IL-12p70 by MDMs. However, the secretion of TNF-α and IL-6 were higher upon stimulation with *A. flavus* AI fraction, and there was significantly higher secretion of IL-8 and IL-12p70 by *A. flavus* AS fraction, compared to the AS fraction of the *A. fumigatus* conidial cell wall.

**Figure 7:**
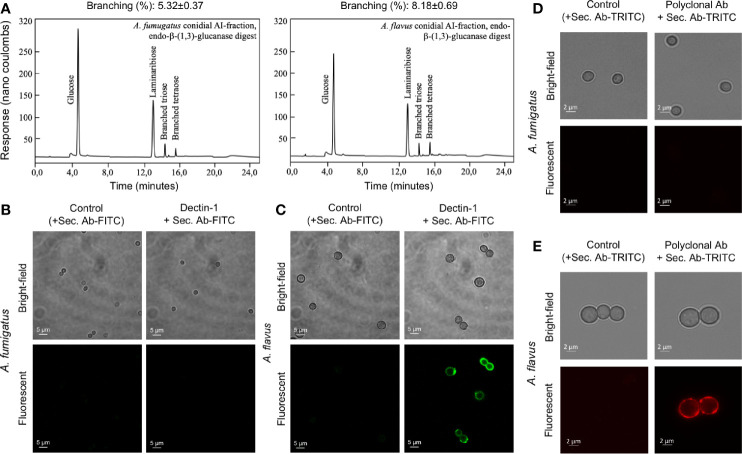
The cell wall β-(1,3)-glucan, branching and exposure on the conidial surfaces of two *Aspergillus* species. **(A)** Conidial alkali-insoluble (AI) fractions of the two *Aspergillus* species were digested with endo-β-(1,3)-glucanase (LamA) and the solubilized materials were subjected to anion-exchange chromatography (DIONEX). β-(1,3)-Glucan branching percentages were calculated based on the peak areas in the chromatography profiles. **(B)** *A. fumigatus* and **(C)** *A. flavus* conidia were labelled for β-(1,3)-glucan with human-Fc conjugated Dectin-1 and anti-human-Fc-specific IgG-FITC; conidia treated by only secondary antibody served as the controls. **(D)** *A. fumigatus* and **(E)** *A. flavus* conidia were labelled for α-(1,3)-glucan with mouse polyclonal antibody specific for α-(1,3)-glucan and anti-mouse IgG-TRITC; conidia treated by secondary antibody served as the controls.

Differential stimulation of the protein extracts from two *Aspergillus* species could be attributed to their protein compositions (**Supplementary Table S1**). Among polysaccharide ensembles, galactomannan content in the conidial cell wall fractions of the two *Aspergillus* species were comparable (**Figure 5A**). Therefore, we hypothesized that the differential stimulation of MDMs by their cell wall fractions could be due to other polysaccharides. In the AI fractions, there was a significant difference in the chitin contents of the two species. However, chitinase digestion of the AI fraction resulted in similar anion-exchange chromatography profiles, suggesting their structural similarity. On the other hand, although the β-1,3-glucan amounts were comparable, β-1,3-glucanase digestion of the AI fraction showed a significant difference in branching for the two *Aspergillus* species (**Figure 7A**). β-1,3-glucan of *A. flavus* showed ˜1.5-fold higher percent of branching compared to that in *A. fumigatus* (8.18 ± 0.69% and ˜5.32 ± 0.37%, respectively, for *A. flavus* and *A. fumigatus*). Regarding the AS fractions, there was a significant difference in the α-1,3-glucan content between the two *Aspergillus* species (**Figure 5A**). Further, immunolabeling indicated the exposure of β-1,3-glucan and α-1,3-glucan only on *A. flavus* conidial surface (**Figures 7B–E**). Together, difference in the amount, structure, and organization of the polysaccharides in the cell wall could be responsible the differential immunostimulatory capacities of the conidia of *A. fumigatus* and *A. flavus*.

### Susceptibility to Cell Wall Perturbing Agents and Cell Wall Directed Antifungals Are Not the Same for the Two *Aspergillu*s Species

**Figure 8:**
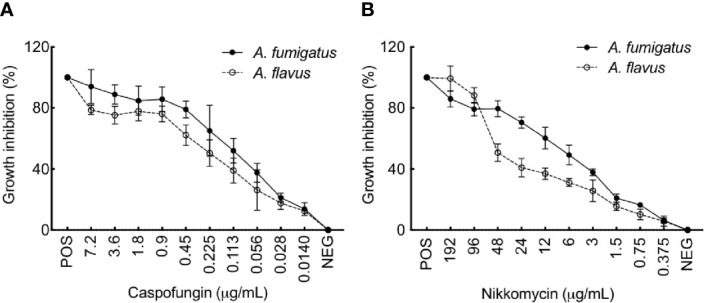
**(A)** *flavus* is more resistant to Caspofungin and Nikkomycin compared to *A. fumigatus*. Growth inhibition of *A. fumigatus* and *A. flavus* in the presence of **(A)** Caspofungin and **(B)** Nikkomycin, as assessed by resazurin method [positive control (POS): amphotericin B (AMB; 2 mg per ml, 4 μl per well) and negative control (NEG): without any drug]. Error bar – standard deviation.

We then examined the effect of cell wall perturbing agents, Congo red (CR), and Calcofluor white (CFW), on the growth of the two *Aspergillus* species on solid agar medium (**Supplementary Figure 6A**). With increasing concentration of CR (50–250 µg/ml), a growth retardation was observed for *A. fumigatus* (40% reduction in the radial growth at 250 µg/ml), while *A. flavus* displayed only 10% growth retardation at 250 µg/mL of CR. The different concentration of CFW tested (50-250 µg/mL) could inhibit the radial growth of *A. flavus* up to 10%–30%, while *A. fumigatus* showed 80% reduction in the radial growth at 150 µg/ml and it failed to grow above 200 µg/ml of CFW. Menadione and H_2_O_2_, the oxidative stress inducing agents, did not inhibit the growth of *A. flavus*, whereas the growth of *A. fumigatus* was inhibited with the highest concentrations of the menadione tested (80-160 µM; **Supplementary Figure 6B**). We further investigated the sensitivity of the two *Aspergillus* species toward caspofungin and nikkomycin, the antifungals targeting membrane bound β-1,3-glucan and chitin biosynthesis, respectively. *A. flavus* showed lower sensitivity towards both caspofungin and nikkomycin, compared to *A. fumigatus* (**Figure 8**). *A. flavus* was susceptible to nikkomycin only at concentrations >96 μg/ml.

## Discussion

In this study, we demonstrate that *A. fumigatus* and *A. flavus* conidia with different sizes, cell surface architectures and cell wall compositions, interact differentially with the host immune system. *A. flavus* conidia were heterogeneous in sizes, with surface exposed polysaccharides, and an amorphous layer interfacing the surface rodlet layer. In contrary, *A. fumigatus* conidia were homogenous in size with rodlet layer covering their surface. *A. flavus* conidia contained more fibrillar polysaccharides in their cell-wall compared to *A. fumigatus* conidia. With all these architectural and compositional differences, the conidia of *A. flavus*, even when dormant, were immunostimulatory, in contrast to *A. fumigatus* conidia that were immunologically inert (Aimanianda et al., 2009). The phenotypic and compositional differences observed in the cell wall between *A. fumigatus* and *A. flavus* were validated using a panel of clinical isolates recovered from the different sites of infection for each species.

Airborne pathogen size and its hydrophobicity dictate the extent of its entry into the respiratory tract (Gralton et al., 2011; Fernstrom and Goldblatt, 2013). We found that *A. flavus* conidia are bigger in size and heterogeneous as reported earlier (Sweany et al., 2011; Goncalves et al., 2012; Runa et al., 2015). *A. fumigatus* conidia were uniformly covered by a hydrophobic rodlet layer, while *A. flavus* conidia showed additional amorphous surfaces patches that contribute to lowering the conidial hydrophobicity. A slight decline in surface hydrophobicity of the fungal conidia reduces the efficiency of aerial dispersal, as demonstrated for *Trichoderma* conidia (Cai et al., 2020). Being smaller in size and hydrophobic, *A. fumigatus* conidia are possibly more efficient than *A. flavus* conidia in aerial dispersal and easily enter the deeper part of the respiratory system. Indeed, we observed that the conidial number has an impact on the virulence of *A. flavus* in our immunocompromised mice model. On the other hand, the risk factor of fungal keratitis is ocular trauma (Niu et al., 2020), which creates hydrophilic surface for direct conidial contact. *A. flavus* has been reported to cause spectrum of human infections prevalently in Asia, Africa and Middle Eastern countries. The reasons have been attributed to its survival in hot and arid climatic conditions in comparison with other *Aspergillus* species (Rotjanapan et al., 2018; Rudramurthy et al., 2019). In contrast, we observed optimal growth for both *A. fumigatus* and *A. flavus* at 37°C; however, *A. fumigatus* showed growth even at higher temperature. Whereas, *A. flavus* showed a faster germination rate in liquid medium, compared to *A. fumigatus*.

Conidial cell wall being the first to interact with the host immune system, its surface organization and composition potentially play a role in the immunomodulation of the host immune system. Earlier, we demonstrated that the *A. fumigatus* conidia surface rodlet layer masks conidial recognition by the host immune system (Aimanianda et al., 2009). Unlike *A. fumigatus* conidia, there were surface exposed antigenic polysaccharides and discontinuous rodlet layer on *A. flavus* conidial surfaces. This prompted use to examine the conidial cell wall organization of the two *Aspergillus* species. Mass-spectrometric analysis of the conidial cell wall protein extract revealed that the RodA PSM score was the highest for *A. fumigatus*, but not for *A. flavus*; in *A. flavus*, the highest PSM score was observed for a putative GPI-anchored protein (UniProt accession No.: B8N327). *A. fumigatus* RodA was originally predicted to be a GPI-anchored protein. We therefore BLASTed this putative GPI anchored protein against the protein sequences in UniProt, but it was not matched with any hydrophobin.

*A. flavus* conidia were decorated with surface exposed polysaccharides (β-1,3-glucan, chitin, α-1,3-glucan, and galactomannan), and contained more fibrillar polysaccharides in their cell wall compared to *A. fumigatus* conidia. These architectural and compositional differences could be responsible for the immunostimulatory potential of *A. flavus* conidia in contrast to immunologically inert *A. fumigatus* conidia (Aimanianda et al., 2009). Despite being dormant, *A. flavus* conidia stimulated TNF-α, IFN-γ, IL-1β, IL-2, IL-6, IL-8, IL-12p70, and IL-10 secretion from MDMs and IL-8 secretion from neutrophils and DCs. This Th1-type response represented by the stimulation of proinflammatory cytokines favors elimination of the fungal pathogen (Cenci et al., 1997). For e.g., IL-8 induces respiratory burst in neutrophils (Baggiolini et al., 1989), while neutralization of TNF-α lowers survival rate in the animal model of invasive pulmonary aspergillosis (Mehrad et al., 1999). Significantly higher secretion of CXCL1/KC, IL-1β, and IL-6 in the mouse lung upon infection with *A. flavus* conidia also suggest that *A. flavus* conidia are more efficiently cleared by the host as compared to *A. fumigatus* conidia.

Even though the cell wall β-1,3-glucan amount was similar in the conidia of two *Aspergillus* species, β-1,3-glucan of *A. flavus* was more branched compared to that of *A. fumigatus*. Branched β-1,3-glucan is more accessible to immune cells as linear β-1,3-glucan forms fibrillar/particulate structure (Camilli et al., 2018). Chitin has been demonstrated to be sensed by the host immune system, activating innate and adaptive immune cells (Elieh Ali Komi et al., 2018). Higher amount of chitin in the cell wall and its surface exposure on *A. flavus* conidia may explain its better recognition by alveolar macrophages. We demonstrated that α-1,3-glucan induces the secretion of TNF-α, IL-1β, IL-6, and IL-10 (Stephen-Victor et al., 2017). The surface exposure of α-1,3-glucan in *A. flavus* conidia, therefore, may also contribute to the cytokine secretion. Interestingly, GAG was found localized in the fibrillar fraction of the cell wall in the *A. flavus* germ tubes, contrast to *A. fumigatus* wherein GAG is exposed on the germ tube surface (Fontaine et al., 2011). GAG increases the fungal virulence *via* resistance to neutrophil extracellular traps (NETs), a host defense mechanism (Lee et al., 2015). Thus, the non-exposed GAG in germ tubes suggest that *A. flavus* is more susceptible to NETs compared to *A. fumigatus* germ tubes.

*A. flavus* has been reported to be resistant to polyenes, possibly by (i) the over-expression of efflux pump leading to high minimum inhibitory concentrations (MIC), (ii) due to the higher level of ergosterol in their cell membrane, or (iii) increased peroxidase activity resulting in lower peroxidation of membrane lipids (Rudramurthy et al., 2019). Azole-resistance and underlying mechanism are well documented for both *Aspergillus* species (Meis et al., 2016; Verweij et al., 2016; Chowdhary et al., 2017; Sharma et al., 2018). However, the roles of molecular differences associated with both *A. fumigatus* and *A. flavus* cell wall in their differential drug-susceptibility remains to be determined. In our study, germinating *A. flavus* showed lower amount of β-1,3-glucan and higher amounts of chitin and GAG in the mycelial cell wall compared to *A. fumigatus.* In correlation, compared to *A. fumigatus*, *A. flavus* was less sensitive to both caspofungin (a β-1,3-glucan synthase inhibitor) and nikkomycin (a chitin synthase inhibitor), suggesting that the drugs targeting these cell wall polysaccharides may not be effective in treatment against invasive infection caused because of *A. flavus*.

Overall, differential organization and composition of the conidial cell wall of *A. fumigatus* and *A. flavus* may affect their specific interaction with host immune system. This could play essential roles in disease establishing potentials of these two *Aspergillus* species. Accordingly, infections caused by them may require different antifungal drug treatment strategies. Nonetheless, these *Aspergillus* species are also known to secrete secondary metabolites, which have not been explored in our study and should be addressed in the future with regard to their virulence and the disease establishment. Moreover, it will be of interest to study the role played by the *A. flavus* conidial sizes in their capacity toward stress-resistance and their interaction with host immune system.

## Data Availability Statement

Mass spectrometry proteomics data obtained in this study have been deposited to the ProteomeXchange Consortium (Vizcaino et al., 2014) via the PRIDE partner repository with the dataset identifier PXD020592 with the username: reviewer99298@ebi.ac.uk and the password: cvTTje6T.

## Ethics Statement

Blood samples from healthy donors were obtained from Etablissement Français du Sang (Saint-Louis hospital, Paris, France) with written and informed consent as per the guidelines provided by the institutional ethics committee, Institut Pasteur (convention 12/EFS/023). Institut National de la Santé et de la Recherche-EFS ethical committee permission (N°18/EFS/033) has been obtained for the use of human samples. The patients/participants provided their written informed consent to participate in this study. The animal experiments were approved by the ethical committee for animal experimentation Comité d’Éthique en Experimentation Animale (CETEA Project license number 2013-0055).

## Author Contributions

LP, JM, KD, J-PL, and VA conceived and designed this study. SW, LV, AB, AK, MS, JJ, JB, and VA performed the experiments and analyses of the data. SB provided critical conceptual inputs. VA drafted the manuscript. All authors contributed to the article and approved the submitted version.

## Funding

This work, including a postdoctoral fellowship to SW and a project assistantship to LV, was supported by the Centre Franco-Indien pour la Promotion de la Recherche Avancée (CEFIPRA) grant No. 5403-1. VA was also supported by ANR-FUNHYDRO (ANR-16S-CE110020-01) grant.

## Conflict of Interest

The authors declare that the research was conducted in the absence of any commercial or financial relationships that could be construed as a potential conflict of interest.

## References

[B1] Aimanianda V. Bayry J. Bozza S. Kniemeyer O. Perruccio K. Elluru S. R.. (2009). Surface hydrophobin prevents immune recognition of airborne fungal spores. Nature 460, 1117–1121. 10.1038/nature08264 19713928

[B2] Akpek E. K. Gottsch J. D. (2003). Immune defense at the ocular surface. Eye 17, 949–956. 10.1038/sj.eye.6700617 14631402

[B3] Al-Hatmi A. M. S. Castro M. A. De Hoog G. S. Badali H. Alvarado V. F. Verweij P. E.. (2019). Epidemiology of Aspergillus species causing keratitis in Mexico. Mycoses 62, 144–151. 10.1111/myc.12855 30256460

[B4] Alsteens D. Aimanianda V. Hegde P. Pire S. Beau R. Bayry J.. (2013). Unraveling the nanoscale surface properties of chitin synthase mutants of Aspergillus fumigatus and their biological implications. Biophys. J. 105, 320–327. 10.1016/j.bpj.2013.05.040 23870253 PMC3714923

[B5] Amaike S. Keller N. P. (2011). Aspergillus flavus. Annu. Rev. Phytopathol. 49, 107–133. 10.1146/annurev-phyto-072910-095221 21513456

[B6] Ansari Z. Miller D. Galor A. (2013). Current thoughts in fungal keratitis: Diagnosis and treatment. Curr. Fungal Infect. Rep. 7, 209–218. 10.1007/s12281-013-0150-1 24040467 PMC3768010

[B7] Baggiolini M. Walz A. Kunkel S. (1989). Neutrophil-activating peptide-1/interleukin 8, a novel cytokine that activates neutrophils. J. Clin. Invest. 84, 1045–1049. 10.1172/JCI114265 2677047 PMC329758

[B8] Bongomin F. Gago S. Oladele R. O. Denning D. W. (2017). Global and Multi-National Prevalence of Fungal Diseases-Estimate Precision. J. Fungi (Basel) 3. 10.3390/jof3040057 PMC5753159 29371573

[B9] Cai F. Gao R. Zhao Z. Ding M. Jiang S. Yagtu C.. (2020). Evolutionary compromises in fungal fitness: hydrophobins can hinder the adverse dispersal of conidiospores and challenge their survival. Isme. J. 14, 2610–2624. 10.1038/s41396-020-0709-0 32632264 PMC7490268

[B10] Camilli G. Eren E. Williams D. L. Aimanianda V. Meunier E. Quintin J. (2018). Impaired phagocytosis directs human monocyte activation in response to fungal derived beta-glucan particles. Eur. J. Immunol. 48, 757–770. 10.1002/eji.201747224 29313961 PMC6291007

[B11] Cenci E. Perito S. Enssle K. H. Mosci P. Latge J. P. Romani L.. (1997). Th1 and Th2 cytokines in mice with invasive aspergillosis. Infect. Immun. 65, 564–570. 10.1128/IAI.65.2.564-570.1997 9009314 PMC176097

[B12] Chowdhary A. Sharma C. Meis J. F. (2017). Azole-resistant aspergillosis: Epidemiology, molecular mechanisms, and treatment. J. Infect. Dis. 216, S436–s444. 10.1093/infdis/jix210 28911045

[B13] Clavaud C. Beauvais A. Barbin L. Munier-Lehmann H. Latge J. P. (2012). The composition of the culture medium influences the beta-1,3-glucan metabolism of Aspergillus fumigatus and the antifungal activity of inhibitors of beta-1,3-glucan synthesis. Antimicrob. Agents Chemother. 56, 3428–3431. 10.1128/AAC.05661-11 22391552 PMC3370737

[B14] Cole D. C. Govender N. P. Chakrabarti A. Sacarlal J. Denning D. W. (2017). Improvement of fungal disease identification and management: combined health systems and public health approaches. Lancet Infect. Dis. 17, e412–e419. 10.1016/S1473-3099(17)30308-0 28774694

[B15] Denning D. W. Bromley M. J. (2015). Infectious Disease. How to bolster the antifungal pipeline. Science 347, 1414–1416. 10.1126/science.aaa6097 25814567

[B16] Dogra V. Sinha A. K. Saxena R. Talwar D. (2016). Aspergillus march: from ABPA to aspergilloma to subacute invasive aspergillosis. Allergy Asthma Clin. Immunol. 12, 64. 10.1186/s13223-016-0170-9 27980539 PMC5135745

[B17] Elieh Ali Komi D. Sharma L. Dela Cruz C. S. (2018). Chitin and its effects on inflammatory and immune responses. Clin. Rev. Allergy Immunol. 54, 213–223. 10.1007/s12016-017-8600-0 28251581 PMC5680136

[B18] Fernstrom A. Goldblatt M. (2013). Aerobiology and its role in the transmission of infectious diseases. J. Pathog. 2013, 493960. 10.1155/2013/493960 23365758 PMC3556854

[B19] Fontaine T. Delangle A. Simenel C. Coddeville B. Van Vliet S. J. Van Kooyk Y.. (2011). Galactosaminogalactan, a new immunosuppressive polysaccharide of Aspergillus fumigatus. PloS Pathog. 7, e1002372. 10.1371/journal.ppat.1002372 22102815 PMC3213105

[B20] Geiser D. M. (2009). Sexual structures in Aspergillus: morphology, importance and genomics. Med. Mycol. 47 (Suppl 1), S21–S26. 10.1080/13693780802139859 18608901

[B21] Goncalves S. S. Cano J. F. Stchigel A. M. Melo A. S. Godoy-Martinez P. C. Correa B.. (2012). Molecular phylogeny and phenotypic variability of clinical and environmental strains of Aspergillus flavus. Fungal Biol. 116, 1146–1155. 10.1016/j.funbio.2012.08.006 23153805

[B22] Gralton J. Tovey E. Mclaws M. L. Rawlinson W. D. (2011). The role of particle size in aerosolised pathogen transmission: a review. J. Infect. 62, 1–13. 10.1016/j.jinf.2010.11.010 21094184 PMC7112663

[B23] Jimenez-Ortigosa C. Aimanianda V. Muszkieta L. Mouyna I. Alsteens D. Pire S.. (2012). Chitin synthases with a myosin motor-like domain control the resistance of Aspergillus fumigatus to echinocandins. Antimicrob. Agents Chemother. 56, 6121–6131. 10.1128/AAC.00752-12 22964252 PMC3497188

[B24] Kandhavelu J. Demonte N. L. Namperumalsamy V. P. Prajna L. Thangavel C. Jayapal J. M.. (2017). Aspergillus flavus induced alterations in tear protein profile reveal pathogen-induced host response to fungal infection. J. Proteomics 152, 13–21. 10.1016/j.jprot.2016.10.009 27789337

[B25] Lee M. J. Liu H. Barker B. M. Snarr B. D. Gravelat F. N. Al Abdallah Q.. (2015). The Fungal Exopolysaccharide Galactosaminogalactan Mediates Virulence by Enhancing Resistance to Neutrophil Extracellular Traps. PloS Pathog. 11, e1005187. 10.1371/journal.ppat.1005187 26492565 PMC4619649

[B26] Leema G. Kaliamurthy J. Geraldine P. Thomas P. A. (2010). Keratitis due to Aspergillus flavus: clinical profile, molecular identification of fungal strains and detection of aflatoxin production. Mol. Vis. 16, 843–854. 20461152 PMC2866576

[B27] Maddur M. S. Sharma M. Hegde P. Stephen-Victor E. Pulendran B. Kaveri S. V.. (2014). Human B cells induce dendritic cell maturation and favour Th2 polarization by inducing OX-40 ligand. Nat. Commun. 5, 4092. 10.1038/ncomms5092 24910129 PMC4388556

[B28] Mehrad B. Strieter R. M. Standiford T. J. (1999). Role of TNF-alpha in pulmonary host defense in murine invasive aspergillosis. J. Immunol. 162, 1633–1640. 9973423

[B29] Meis J. F. Chowdhary A. Rhodes J. L. Fisher M. C. Verweij P. E. (2016). Clinical implications of globally emerging azole resistance in Aspergillus fumigatus. Philos. Trans. R. Soc. Lond. B. Biol. Sci. 371. 10.1098/rstb.2015.0460 PMC5095539 28080986

[B30] Mouyna I. Aimanianda V. Hartl L. Prevost M. C. Sismeiro O. Dillies M. A.. (2016). GH16 and GH81 family beta-(1,3)-glucanases in Aspergillus fumigatus are essential for conidial cell wall morphogenesis. Cell Microbiol. 18, 1285–1293. 10.1111/cmi.12630 27306610

[B31] Niu L. Liu X. Ma Z. Yin Y. Sun L. Yang L.. (2020). Fungal keratitis: Pathogenesis, diagnosis and prevention. Microb. Pathog. 138, 103802. 10.1016/j.micpath.2019.103802 31626916

[B32] Oliveira M. Pereira C. Bessa C. Araujo R. Saraiva L. (2015). Chronological aging in conidia of pathogenic Aspergillus: Comparison between species. J. Microbiol. Methods 118, 57–63. 10.1016/j.mimet.2015.08.021 26341609

[B33] Palomar A. P. D. Montolio A. Cegonino J. Dhanda S. K. Lio C. T. Bose T. (2019). The Innate Immune Cell Profile of the Cornea Predicts the Onset of Ocular Surface Inflammatory Disorders. J. Clin. Med. 8. 10.3390/jcm8122110 PMC6947418 31810226

[B34] Rotjanapan P. Chen Y. C. Chakrabarti A. Li R. Y. Rudramurthy S. M. Yu J.. (2018). Epidemiology and clinical characteristics of invasive mould infections: A multicenter, retrospective analysis in five Asian countries. Med. Mycol. 56, 186–196. 10.1093/mmy/myx029 28525619

[B35] Rudramurthy S. M. Paul R. A. Chakrabarti A. Mouton J. W. Meis J. F. (2019). Invasive Aspergillosis by Aspergillus flavus: Epidemiology, Diagnosis, Antifungal Resistance, and Management. J. Fungi (Basel) 5. 10.3390/jof5030055 PMC6787648 31266196

[B36] Runa F. Carbone I. Bhatnagar D. Payne G. A. (2015). Nuclear heterogeneity in conidial populations of Aspergillus flavus. Fungal Genet. Biol. 84, 62–72. 10.1016/j.fgb.2015.09.003 26362651

[B37] Sharma C. Kumar R. Kumar N. Masih A. Gupta D. Chowdhary A. (2018). Investigation of Multiple Resistance Mechanisms in Voriconazole-Resistant Aspergillus flavus Clinical Isolates from a Chest Hospital Surveillance in Delhi, India. Antimicrob. Agents Chemother. 62, e01928-17. 10.1128/AAC.01928-17 PMC5826139 29311090

[B39] Stephen-Victor E. Karnam A. Fontaine T. Beauvais A. Das M. Hegde P.. (2017). Aspergillus fumigatus Cell Wall alpha-(1,3)-Glucan Stimulates Regulatory T-Cell Polarization by Inducing PD-L1 Expression on Human Dendritic Cells. J. Infect. Dis. 216, 1281–1294. 10.1093/infdis/jix469 28968869

[B40] Suzuki T. Chow C. W. Downey G. P. (2008). Role of innate immune cells and their products in lung immunopathology. Int. J. Biochem. Cell Biol. 40, 1348–1361. 10.1016/j.biocel.2008.01.003 18272422

[B41] Sweany R. R. Damann K. E. Jr. Kaller M. D. (2011). Comparison of soil and corn kernel Aspergillus flavus populations: evidence for niche specialization. Phytopathology 101, 952–959. 10.1094/PHYTO-09-10-0243 21405994

[B42] Tekaia F. Latge J. P. (2005). Aspergillus fumigatus: saprophyte or pathogen? Curr. Opin. Microbiol. 8, 385–392. 10.1016/j.mib.2005.06.017 16019255

[B43] Thau N. Monod M. Crestani B. Rolland C. Tronchin G. Latge J. P.. (1994). rodletless mutants of Aspergillus fumigatus. Infect. Immun. 62, 4380–4388. 10.1128/IAI.62.10.4380-4388.1994 7927699 PMC303120

[B44] Valsecchi I. Stephen-Victor E. Wong S. S. W. Karnam A. Sunde M. Guijarro J. I.. (2020). The Role of RodA-Conserved Cysteine Residues in the Aspergillus fumigatus Conidial Surface Organization. J. Fungi (Basel) 6. 10.3390/jof6030151 PMC7558875 32859091

[B45] Verweij P. E. Chowdhary A. Melchers W. J. Meis J. F. (2016). Azole resistance in Aspergillus fumigatus: Can we retain the clinical use of mold-active antifungal azoles? Clin. Infect. Dis. 62, 362–368. 10.1093/cid/civ885 26486705 PMC4706635

[B46] Vizcaino J. A. Deutsch E. W. Wang R. Csordas A. Reisinger F. Rios D.. (2014). ProteomeXchange provides globally coordinated proteomics data submission and dissemination. Nat. Biotechnol. 32, 223–226. 10.1038/nbt.2839 24727771 PMC3986813

[B47] Wong S. S. W. Rani M. Dodagatta-Marri E. Ibrahim-Granet O. Kishore U. Bayry J.. (2018). Fungal melanin stimulates surfactant protein D-mediated opsonization of and host immune response to Aspergillus fumigatus spores. J. Biol. Chem. 293, 4901–4912. 10.1074/jbc.M117.815852 29414772 PMC5880149

