# Supplementary material for: Species-Specific Immunological Reactivities Depend on the Cell-Wall Organization of the Two Aspergillus, Aspergillus fumigatus and A. flavus
Source: Front Cell Infect Microbiol. 2021 Feb 25;11:643312. doi: 10.3389/fcimb.2021.643312 (PMC7950546; doi:10.3389/fcimb.2021.643312)
Supplement: Supplementary file 1 [file DataSheet_1.pdf]

Supplementary Information

Supplementary Table S1A: Proteins identified in the formic acid extract of *Aspergillus fumigatus* conidia

UniProt accession	Description	Unique peptides	PSMs	M_W [kDa]	*Cell surface
P41746	Hydrophobin	9	495	16,1	Yes
Q4WPK6	GPI anchored protein, putative	13	374	102,8	?
E9QT94	Hydrophobin	5	188	14,3	Yes
Q4WJJ3	Probable beta-glucosidase A	24	183	94,7	Yes
Q4WRH9	Probable alpha/beta-glucosidase agdC	33	180	98,8	Yes
Q92405	Catalase B	26	168	79,9	Yes
Q4WV25	ATP synthase subunit beta	27	148	55,6	No
O42630	Vacuolar protease A	14	146	43,3	Yes
Q4WK60	Probable glucan 1,3-beta-glucosidase A	15	144	45,7	Yes
Q4WZ26	Adenosine deaminase family protein	18	135	66,2	?
Q4WCB5	Beta-hexosaminidase	21	127	67,4	?
Q4WMU2	Alkaline phosphatase, putative	18	119	71,8	?
A4D9F7	Class V chitinase, putative	20	110	46,5	Yes
Q4WB00	Methyltransferase psoC	16	102	29,5	?
E9R226	Exo-beta-1,3-glucanase Exg0	16	98	100,6	Yes
A4DA60	Purple acid phosphatase	16	98	54,0	?
Q4WYS0	Extracellular phytase, putative	23	97	72,5	?
Q4WBR8	Hydrophobin	4	91	16,0	Yes
Q4WH99	Protein disulfide-isomerase	22	86	56,2	No
P87184	Alkaline protease 2	10	85	52,6	Yes
Q4WX47	Alpha-mannosidase	26	83	123,7	No
Q4WGT3	Probable beta-glucosidase L	19	83	78,3	Yes
P0C959	Dipeptidyl-peptidase 5	19	80	79,7	Yes
E9RBR0	Laccase abr2	12	79	65,2	Yes
Q4WT26	Alpha-1,2-mannosidase family protein, putative	19	76	92,7	?
Q4WIT8	Glycosyl hydrolase, putative	10	75	40,9	?
Q4WRZ5	Probable mannosyl-oligosaccharide alpha-1,2-mannosidase 1B	17	68	53,8	No
Q70J59	Tripeptidyl-peptidase sed2	16	66	65,8	Yes
Q4WV20	Amidase, putative	18	63	65,0	No
Q4WWT9	Glutaminase GtaA	14	63	76,1	?
Q4WC88	ATP synthase subunit alpha	20	62	59,9	No
Q4WLW6	FAD-linked oxidoreductase fmqD	15	60	54,5	Yes
Q4WJD2	Elongation factor 1-alpha	16	59	53,8	?
Q4WED7	O-methyltransferase, putative	14	58	47,7	?
Q4WCE3	Oligopeptidase family protein	19	56	79,9	?
Q70GH4	Tripeptidyl-peptidase sed3	9	56	65,2	Yes
Q4WK08	FG-GAP repeat protein, putative	11	54	33,7	?
O00092	3-phytase A	13	53	50,8	Yes
Q4WNT7	Outer mitochondrial membrane protein porin	10	53	36,9	No
Q4WGI4	Class III chitinase, putative	4	51	46,2	Yes
Q5VJG9	Carboxypeptidase Y homolog A	11	48	60,9	No
Q4WDX7	Glyceraldehyde-3-phosphate dehydrogenase	10	46	36,1	No
Q4WYX2	Histidine acid phosphatase, putative	11	45	51,3	?

Q4WQH7	Methylmalonate-semialdehyde dehydrogenase, putative	14	44	63,8	No
P28296	Alkaline protease 1	7	43	42,2	Yes
Q4WSY6	Eukaryotic translation elongation factor 1 subunit Eef1-beta, putative	11	42	25,2	No
Q4WJN2	Mitochondrial ADP, ATP carrier protein (Ant), putative	8	42	33,3	No
Q4WLU9	Glycerophosphoryl diester phosphodiesterase family protein	11	41	46,4	?
A0A067Z9B6	O-methyltransferase af390-400	11	39	44,5	?
Q4WL94	Glucooligosaccharide oxidase, putative	12	38	51,4	?
Q4WV22	Alpha-1,2-mannosidase family protein	12	38	89,4	No
Q4WMU3	Probable beta-glucosidase F	11	38	92,9	Yes
Q4WS20	Fasciclin domain family protein	10	38	55,3	Yes
Q4W9U8	Acid phosphatase, putative	9	38	46,1	?
Q4WGZ3	Phosphoglycerate mutase family protein, putative	5	38	20,8	?
Q4WQS0	Exo-alpha-sialidase	12	37	44,4	No
P67875	Ribonuclease mitogillin	6	37	19,6	Yes
Q4WE77	Extracellular lipase, putative	6	36	31,4	?
Q4WI29	14-3-3 family protein ArtA, putative	8	35	29,1	?
E9QV41	Phosphoesterase superfamily protein	8	34	49,9	?
Q4W9C9	Acid phosphatase, putative	7	34	30,4	?
Q4WIE8	Dihydrolipoyl dehydrogenase	10	33	54,9	No
Q4WIN3	Beta-fructofuranosidase, putative	10	32	57,2	No
Q4WEA3	Peroxidase, putative	9	32	30,7	?
E9QRF2	Endochitinase B1	8	32	47,6	Yes
Q4WKB0	ATP synthase delta chain, mitochondrial, putative	5	32	17,6	No
Q4WDJ0	Formate dehydrogenase	12	31	45,7	No
Q4WAG2	Aspergillopepsin, putaitve	3	31	29,0	?
Q4W9Z2	Extracellular guanyl-specific ribonuclease RntA	5	30	14,0	?
Q4X078	Cutinase	5	30	36,4	Yes
Q4WE70	Glyceraldehyde-3-phosphate dehydrogenase	10	29	36,3	No
A4D9D3	Extracellular serine rich protein	5	29	50,5	No
Q4WUL0	Woronin body protein HexA, putative	8	28	61,4	No
Q4WT45	GPI anchored protein, putative	6	28	38,9	No
Q4WD55	IgE-binding protein, putative	3	28	21,2	?
Q4WJC4	Neutral/alkaline nonlysosomal ceramidase, putative	8	27	83,4	Yes
Q4WFW0	FAD-dependent oxygenase, putative	7	27	55,0	?
Q4W930	Probable arabinan endo-1,5-alpha-L-arabinosidase C	5	27	35,0	Yes
Q4WNY2	Cobalamin-independent methionine synthase MetH/D	9	26	86,8	?
Q4WGP3	Malate dehydrogenase	8	26	35,9	No
Q4WMD4	Thioredoxin, putative	7	26	11,9	?
Q4WSQ8	Alternative NADH-dehydrogenase	7	24	68,2	?
O60022	Allergen Asp F15	4	24	15,9	Yes
Q4WEM5	Thioredoxin reductase, putative	9	23	42,8	?
Q4WEU3	Ubiquinol-cytochrome C reductase complex core protein 2, putative	8	22	48,1	No
Q4W962	Glycosyl hydrolase, putative	8	22	98,9	Yes
Q4WKD8	NADH-quinone oxidoreductase Pst2, putative	7	22	21,8	?
Q4WRT7	Mitochondrial phosphate carrier protein (Mir1), putative	6	22	33,8	No
Q4WDH2	Actin Act1	7	20	43,9	Yes
Q4WK69	ATP synthase subunit gamma	5	20	32,4	No
Q4WAE9	Cysteine-rich secreted protein	4	20	36,8	?

Q4WAZ3	Dioxygenase af480	7	18	34,2	?
Q4WJ30	Molecular chaperone Hsp70	7	18	69,6	No
Q4WDZ3	Carboxypeptidase S1, putative	6	18	54,1	No
Q4WKR6	Endonuclease/exonuclease/phosphatase family protein	6	18	47,6	?
Q4WXW3	Glutaminase, putative	5	18	92,7	?
Q4WN06	6-phosphogluconate dehydrogenase, decarboxylating	4	18	55,8	No
Q4WYG0	MFS transporter, putative	3	18	47,8	No
Q4WJZ7	Endo-1,3-beta-glucanase Engl1	8	17	104,9	Yes/No
Q4WQZ2	Glutathione S-transferase-like protein tpcF	6	17	25,0	?
Q4WUP8	Transaldolase	6	17	35,4	No
Q4WSU0	Cytosolic Cu/Zn superoxide dismutase, putative	4	17	24,0	Yes/No
Q4WAZ8	Alpha/beta hydrolase psoB	7	16	49,1	?
Q4WX92	Isocitrate dehydrogenase [NADP]	6	16	55,7	No
Q4WLQ0	Dienelactone hydrolase family protein	6	16	26,9	?
Q4WWY2	Exo-beta-1,3-glucanase, putative	6	16	86,7	Yes
Q4WND4	14-3-3 family protein	5	16	30,1	?
Q4WRE3	Ribonuclease T2, putative	5	16	29,3	Yes
Q4WM72	Extracellular protein, putative	4	16	74,5	?
O42799	Allergen Asp F7	3	16	27,5	?
Q4WXC6	Regulatory protein SUAPRGA1	3	16	40,1	No
Q4WNS8	Protein ecm33	8	15	41,5	Yes/No
Q4WK46	Glutamate carboxypeptidase, putative	7	15	86,1	No
Q4X0G7	Translation elongation factor EF-2 subunit, putative	7	15	93,1	No
Q4WDF5	Translation elongation factor eEF-1 subunit gamma, putative	6	15	54,1	No
A4D9V8	Cupin domain protein	4	15	21,7	?
Q4X1A4	Mannitol-1-phosphate 5-dehydrogenase	7	14	43,0	No
O60024	Allergen Asp F4	6	14	34,1	Yes
Q4WY00	Class V chitinase, putative	6	14	49,1	?
Q4WX43	ATP-dependent RNA helicase eIF4A	5	14	45,7	No
Q4WAZ5	Acetate-CoA ligase, putative	5	14	79,4	?
Q4WQX8	Glutathione S-transferase, putative	5	14	30,0	?
Q4WS30	Mitochondrial processing peptidase beta subunit, putative	5	14	53,2	No
Q4WDM0	Malate dehydrogenase, NAD-dependent	5	14	34,8	No
Q4WX70	Mitochondrial phosphate carrier protein, putative	5	14	44,3	No
Q4X084	Probable endo-1,3(4)-beta-glucanase AFUA_2G14360	4	14	66,0	No
Q4WLT6	Cell wall glucanase (Scw4), putative	4	14	41,0	Yes
Q4X164	Acyl CoA binding protein family	3	14	17,3	?
Q4WR90	Translation elongation factor eEF-1B gamma subunit, putative	3	14	24,4	No
Q4WSA0	Transketolase	5	13	74,8	No
Q4WSV9	1,3-beta-glucanosyltransferase Bgt1	6	12	33,1	Yes/No
P0C7S9	1,3-beta-glucanosyltransferase gel1	5	12	48,0	Yes/No
Q4WSP5	Glycine cleavage system H protein	5	12	18,6	No
Q4WYS3	Palmitoyl-protein thioesterase	5	12	37,5	No
Q4WED9	Pyoverdine/dityrosine biosynthesis protein, putative	5	12	85,8	?
Q4WEP7	Endochitinase A1	4	12	88,6	Yes/No
Q9UUZ6	60S acidic ribosomal protein P2	4	12	11,1	No
Q4WMF0	N,O-diacetyl muramidase, putative	4	12	24,6	?
Q4X1U4	Mitochondrial inner membrane nuclease Nuc1, putative	4	12	37,0	No

Q4W9P4	Leucine aminopeptidase 1	3	12	43,1	Yes
E9R013	Carboxypeptidase	3	12	68,3	No
Q4WX38	Cell wall protein, putative	3	12	60,1	?
Q4WEE8	Cytochrome c oxidase subunit Va, putative	5	11	18,0	No
E9QYY1	Isocitrate dehydrogenase [NAD] subunit, mitochondrial	5	11	41,7	No
Q8X176	Acid phosphatase	3	11	49,0	No
O43099	Peroxiredoxin Asp F3	5	10	18,4	No
Q96X30	Enolase	5	10	47,3	No
Q4WC76	Mitochondrial ATPase subunit ATP4, putative	5	10	29,7	No
Q4WAZ0	Dual-functional monooxygenase/methyltransferase psoF	4	10	101,0	?
Q4WLV6	FK506-binding protein 1A	4	10	12,1	No
Q4WY97	Glutathione peroxidase	4	10	25,7	?
Q4X1H5	Mitochondrial Hsp70 chaperone (Ssc70), putative	4	10	74,4	No
Q4WLJ9	Exo-beta-1,3-glucanase, putative	4	10	84,1	Yes
Q7Z8P9	Nucleoside diphosphate kinase	3	10	16,9	?
Q9HGV0	60S acidic ribosomal protein P1	3	10	11,1	No
Q4WB38	MFS multidrug transporter, putative	3	10	62,8	No
Q4WE17	Allergen, putative	2	10	21,9	No
Q4WGP1	Acetyltransferase component of pyruvate dehydrogenase complex	2	10	52,0	No
Q4WPQ1	Calmodulin	2	9	17,0	No
Q4WGL5	Secreted beta-glucosidase sun1	4	8	43,5	Yes
Q4WT14	Tubulin beta chain	4	8	51,8	No
Q4WYW4	Ketol-acid reductoisomerase	4	8	56,3	No
Q4WT91	Adenosylhomocysteinase	4	8	48,5	No
Q4WPH9	Probable dipeptidyl peptidase 4	3	8	85,8	Yes
Q4WRW6	Acyl-CoA dehydrogenase, putative	3	8	47,4	?
Q4WES0	Vacuolar aspartyl aminopeptidase Lap4, putative	3	8	56,0	No
Q4WX84	Glycine-rich RNA-binding protein, putative	3	8	12,8	No
Q4WPY9	3-ketoacyl-coA thiolase peroxisomal A	3	8	43,5	No
Q4WYJ3	Aldose 1-epimerase, putative	3	8	50,7	No
Q4WHP9	Hsp70 chaperone BiP/Kar2, putative	3	8	73,3	No
Q92450	Superoxide dismutase [Mn], mitochondrial	2	8	23,4	No
Q4WQU0	Tripeptidyl-peptidase sed4	2	8	63,9	Yes
Q4WFS2	Probable aspartic-type endopeptidase AFUA_3G01220	2	8	46,3	Yes
Q4WV27	Cofilin	2	8	17,0	No
Q4WJB3	High expression lethality protein Hel10, putative	2	8	21,8	No
Q4WDN5	Integral membrane protein	2	8	30,4	No
Q4WEY2	Ser/Thr protein phosphatase family	2	8	71,3	No
Q4WB03	Glutathione S-transferase psoE	4	7	26,8	No
Q4WPA3	Mitochondrial peroxiredoxin Prx1, putative	2	7	23,4	No
Q4WKL3	Cytochrome C1/Cyt1, putative	3	6	35,1	No
Q4WPB8	Short chain dehydrogenase, putative	3	6	30,9	?
Q4WG12	Extracellular phytase, putative	3	6	58,3	?
Q4X136	Phosphatidylglycerol/phosphatidylinositol transfer protein	2	6	19,1	?
Q4WXZ5	Ribonuclease T2-like	2	6	45,5	Yes/No
Q4WRV9	Probable Xaa-Pro aminopeptidase AFUA_1G14920	2	6	54,5	?
Q4WHY9	Peptidyl-prolyl cis-trans isomerase	2	6	22,3	No
E9R5X9	Cell wall serine-threonine-rich galactomannoprotein Mp1	2	6	27,3	Yes

Q4WX65	Inorganic diphosphatase, putative	2	6	43,6	No
Q4WPT1	Polyubiquitin UbiD/Ubi4, putative	2	6	35,2	No
Q4WTJ3	60S ribosomal protein L9, putative	2	6	21,8	No
Q4WV97	Thioredoxin	2	6	12,0	?
Q4WXR8	Nuclear transport factor NTF-2, putative	2	6	14,2	No
Q4WQ18	Carbonic anhydrase	2	6	30,8	?
Q4WHT0	Argininosuccinate synthase	2	6	46,1	No
Q4WA10	Profilin	2	6	14,5	No
Q4WK48	UPF0136 domain protein	2	6	16,9	No
E9R8Y4	Phosphatidylglycerol specific phospholipase C, putative	2	6	50,2	?
Q4WTD7	Mitochondrial 2-oxodicarboxylate carrier protein, putative	2	6	33,2	No
Q4WE72	BYS1 domain protein, putative	2	6	16,0	?
Q4WQK8	G-protein comlpex beta subunit CpcB	2	6	35,0	No
Q4WNH3	Isocitrate dehydrogenase [NAD] subunit, mitochondrial	2	6	49,7	No
Q4WWC5	Histone H2B	2	5	14,9	No
Q4W9K3	Mitochondrial outer membrane protein (Sam50), putative	2	5	59,1	No
Q4WGK4	Cellular morphogenesis protein (Rax2), putative	2	5	127,8	No
Q4WV31	Ubiquinol-cytochrome c reductase iron-sulfur subunit	2	5	32,6	No
Q4WJH4	Vacuolar membrane protease	2	4	106,5	No
Q4WRB8	Translationally-controlled tumor protein homolog	2	4	20,2	No
Q8J0P4	Probable glycosidase crf1	2	4	40,3	Yes/No
Q4WAY4	Polyketide transferase af380	2	4	32,7	?
P40292	Heat shock protein 90	2	4	80,6	Yes/No
Q4WWX5	Peptidyl-prolyl cis-trans isomerase	2	4	17,7	No
Q4X1P0	Antigenic mitochondrial protein HSP60, putative	2	4	61,9	No
Q4X046	Endo-arabinase, putative	2	4	35,6	?
Q4WRZ4	Histidine biosynthesis trifunctional protein	2	4	92,8	No
Q4WYA0	60S ribosomal protein L22, putative	2	4	13,4	No
Q4WFT1	Cell wall protein, putative	2	4	19,3	Yes
Q4WQ02	Acetyl-coenzyme A synthetase	2	4	74,4	?
E9QUZ4	ADP-ribosylation factor, putative	2	4	21,0	No
A4DA65	Asp hemolysin-like protein	2	4	15,8	?
Q4W8Z7	IgE-binding protein	2	4	20,5	?
Q4WCL5	Acetyl-CoA acetyltransferase, putative	2	4	40,9	No
Q4WZI3	Endonuclease/exonuclease/phosphatase family protein	2	4	33,8	?
P61832	Histone H3	2	3	15,3	No
Q4WYK1	40S ribosomal protein S0	2	3	32,1	No
Q4X1G7	Actin cytoskeleton protein (VIP1), putative	2	3	28,2	?
Q4WM26	Aldehyde dehydrogenase AldA, putative	2	3	60,9	?
Q4WKG5	Tubulin alpha chain	2	3	50,0	No
E9R3W5	Spermidine synthase	2	3	33,4	?
Q4WXF4	Serine hydroxymethyltransferase	2	3	51,8	?

*The cellular localization of the proteins was referred to by the gene ontology (cellular component) entries on UniProt
Yes: The proteins are annotated to be on the conidial surface
No: The proteins are annotated at non-cell surface location
? No information available
PSMs: Peptide spectrum matches

Supplementary Table S1B: Proteins identified in the formic acid extract of *Aspergillus flavus* conidia

UniProt accession	Description	Unique peptides	PSMs	MW [kDa]	*Cell surface
B8N327	GPI anchored protein, putative	12	529	99,6	No
B8NU30	Class V chitinase, putative	29	297	47,2	?
B8N151	Probable glucan 1,3-beta-glucosidase A	16	216	44,3	Yes
B8MXV3	Beta-hexosaminidase	21	166	67,5	?
B8NCW0	Exo-beta-1,3-glucanase Exg0	19	120	101,2	Yes
B8NRX2	Probable beta-glucosidase A	22	113	93,4	Yes
B8NLE2	Catalase	19	106	79,8	?
B8NWC9	ATP synthase subunit beta	22	92	55,4	No
B8NCH3	Adenosine deaminase family protein	16	79	65,0	?
B8MVM2	Galactose oxidase, putative	14	79	74,3	?
B8MVX4	Oxalate decarboxylase, putative	16	76	51,8	?
B8N3I0	FG-GAP repeat protein, putative	14	73	34,0	?
B8NQF5	Thioredoxin reductase, putative	12	66	42,4	?
B8MZ47	Extracellular cell wall glucanase Crf1/allergen Asp F9	11	58	44,7	Yes/No
B8NBJ1	Multicopper oxidase/laccase, putative	7	56	68,3	?
B8NQD8	Conidial pigment biosynthesis oxidase Arb2/brown2	7	54	50,9	?
B8NXD7	F5/8 type C domain protein	8	53	54,9	?
B8N417	Probable mannosyl-oligosaccharide alpha-1,2-mannosidase 1B	10	52	56,6	No
B8N1C0	Cysteine-rich secreted protein	12	51	36,0	?
B8NPX3	DUF1237 domain protein	10	51	59,2	?
B8N3J2	Endo-1,3-beta-glucanase Engl1	11	50	97,8	?
B8MXS8	ATP synthase subunit alpha	12	45	59,9	No
B8NTJ8	Hydrophobin	2	45	23,4	Yes
B8N7M5	Class V chitinase ChiB1	9	44	48,4	?
B8N106	Alkaline protease 1	8	44	42,5	Yes
B8NVC9	Zinc-binding dehydrogenase family oxidoreductase	7	44	39,5	?
B8NYI6	Phosphatidylglycerol specific phospholipase, putative	11	43	55,7	No
B8NTV2	Glutaminase GtaA	10	43	76,1	?
B8N3Q4	Class V chitinase, putative	9	42	48,2	No
B8NFC2	GILT family thiol reductase, putative	9	41	28,4	No
B8N5U5	Glutamate carboxypeptidase, putative	8	40	73,3	?
B8MXS2	Extracellular matrix protein, putative	6	40	22,3	?
B8N2Q5	Cutinase	6	39	26,0	Yes
B8N3P1	Aspartic endopeptidase Pep2	9	38	43,1	?
B8N8I5	Glucan 1,3-beta-glucosidase, putative	9	37	46,5	?
B8NA16	GPI-anchored cell wall organization protein Ecm33	7	34	41,4	?
B8NRS8	Mitochondrial ADP,ATP carrier protein (Ant), putative	8	33	33,8	No
B8N2Y6	Glyceraldehyde-3-phosphate dehydrogenase	9	32	36,3	No
B8NJS3	Alpha-1,2-mannosidase family protein, putative	9	32	88,5	?
B8N5M5	Phosphatidylglycerol specific phospholipase C, putative	8	32	52,7	?
B8NLF1	Elongation factor 1-alpha	7	32	50,0	?
B8N8Z4	Extracellular protein, putative	6	32	36,4	?
B8NIP3	1,3-beta-glucanosyltransferase Bgt1	8	31	33,4	Yes/No
B8NAJ8	Tripeptidyl-peptidase (TppA), putative	7	31	50,7	Yes
B8NJ90	Glycerophosphoryl diester phosphodiesterase family protein	8	30	45,7	?

B8N747	Extracellular phytase, putative	9	28	72,6	?
B8NUE0	Autophagic serine protease Alp2	6	28	52,4	?
B8NCN3	Extracellular guanyl-specific ribonuclease RntA	2	27	14,0	?
B8NIA0	Fucose-specific lectin FleA	10	25	34,5	?
B8NE68	Purine nucleoside permease, putative	7	25	43,2	?
B8NUR3	40S ribosomal protein S9	7	25	22,2	No
B8N8T4	Aldehyde dehydrogenase AldA, putative	7	24	53,9	?
B8NQA4	Cytochrome c oxidase subunit Va, putative	6	24	17,9	No
B8NUX0	Exo-beta-1,3-glucanase, putative	6	24	82,9	Yes
B8NPM2	Tripeptidyl peptidase A	7	23	63,5	Yes
B8MWN6	Viral-enhancing factor, putative	7	22	95,9	?
B8NTN4	Woronin body major protein, putative	6	22	56,6	?
B8N0I5	Mannan endo-1,6-alpha-mannosidase	6	22	56,1	No
B8N050	Alpha-mannosidase	8	21	123,4	?
B8NVA9	Histone H2B	4	21	15,0	No
B8NCN4	Cell wall serine-threonine-rich galactomannoprotein Mp1	4	20	26,3	?
B8NNS9	Pantothenate transporter, putative	3	20	18,4	No
B8NBJ4	Probable glucan endo-1,6-beta-glucosidase B	6	19	45,2	Yes
B8NLH9	Allergen Asp F3	5	19	18,6	?
B8NWR5	Lipase 8, putative	4	19	45,6	?
B8NXT0	Ribosomal protein S13p/S18e	6	18	17,9	No
B8MZE3	Mitochondrial phosphate carrier protein (Mir1), putative	5	18	32,7	No
B8NA08	Outer mitochondrial membrane protein porin	5	18	37,0	No
B8NF67	Mono-and diacylglycerol lipase, putative	5	18	33,4	?
B8NWD3	Alpha-1,2-mannosidase family protein	6	17	85,2	?
B8N180	Ribosomal L18ae protein family	5	17	28,5	No
B8N7S7	Probable endo-1,3(4)-beta-glucanase AFLA_105200	5	16	68,4	No
B8N3J9	40S ribosomal protein S8	5	16	22,8	No
B8NI10	Beta-cyclopiazonate dehydrogenase	4	16	50,8	?
B8NJU9	GPI anchored protein, putative	4	16	39,8	?
B8N419	Superoxide dismutase	4	16	23,2	?
B8NDY4	Ribosomal protein L14	4	16	17,0	No
B8NFC1	Phosphatidylserine decarboxylase, putative	2	16	51,6	?
B8N5T3	Conidial hydrophobin RodB/HypB	2	15	14,3	?
B8NSD4	40S ribosomal protein S1	5	14	29,2	No
B8NUD9	Transaldolase	4	14	35,5	No
B8N2S6	Cell wall protein PhiA	4	14	19,4	?
B8NCF7	Acid phosphatase, putative	4	14	46,7	?
B8ND52	Actin Act1	4	14	41,6	Yes/No
B8N270	Peptide hydrolase	4	14	53,5	?
B8NC19	60S ribosomal protein L18	3	14	20,8	No
B8NKN3	Peptidyl-prolyl cis-trans isomerase	3	14	23,5	?
B8NAI8	Chitin binding domain protein Peritrophin-A, putative	2	14	15,2	Yes
B8N8P6	Dipeptidase	5	13	44,3	?
B8NVI7	Aspergillopepsin, putative	4	13	28,4	?
B8ND35	Formate dehydrogenase	5	12	40,1	No
B8NBM3	Probable dipeptidyl-peptidase 5	4	12	80,3	Yes
B8NY84	Lipase 2, putative	4	12	48,2	?

B8NSR3	Alpha-L-rhamnosidase B, putative	4	12	74,1	?
B8N6C7	Elastase, putative	4	12	25,0	?
B8NVX2	O-methyltransferase, putative	4	12	48,3	?
B8NPF9	Protein disulfide-isomerase	4	12	56,4	No
B8MZ85	40S ribosomal protein S22	3	12	14,7	No
B8N0A2	Allergen Asp F4	3	12	34,6	Yes
B8NQ60	Peroxidase, putative	3	12	22,9	?
B8N059	Cell wall protein, putative	3	12	79,1	?
B8NQM0	Glycosyl hydrolase, putative	3	12	40,8	?
B8N077	Extracellular serine-rich protein, putative	3	12	85,9	?
B8NF34	Hydrophobin family protein	2	12	27,8	Yes/No
B8NWT9	Aspartic-type endopeptidase, putative	2	12	45,5	?
B8NXS9	Carboxypeptidase Y homolog A	4	11	60,8	No
B8NVT9	Phytase, putative	4	10	57,2	?
B8NJC1	Peptide hydrolase	4	10	46,2	?
B8MW92	Cell wall integrity signaling protein Lsp1/Pil1, putative	4	10	39,2	?
B8NQF0	Nucleoside diphosphate kinase	3	10	17,0	?
B8MY11	Oligopeptidase family protein	3	10	78,8	?
B8NEY4	1,3-beta-glucanosyltransferase	3	10	48,2	Yes/No
B8NEP7	Sphingomyelin phosphodiesterase	3	10	74,1	?
B8NB36	40S ribosomal protein Rps16, putative	3	10	15,9	No
B8MZ58	Ribosomal protein S28e	3	10	7,7	No
B8NUP9	40S ribosomal protein S4	3	10	29,2	No
B8N975	Serine protease, putative	3	10	25,7	?
B8NV82	60S ribosomal protein L27a, putative	3	10	16,8	No
B8N6A6	60S ribosomal protein L5, putative	3	10	34,6	No
B8NVA8	Histone H2A	2	10	14,1	No
B8NPV3	60S ribosomal protein L37a	2	10	10,1	No
B8NB78	40S ribosomal protein S15, putative	2	10	17,5	No
B8N014	Glycine-rich RNA-binding protein, putative	2	10	13,6	?
B8NFW3	Acyl CoA binding protein family	4	9	15,6	?
B8N4Q2	Histone H3	3	9	15,3	No
B8N0Z6	Peptidyl-prolyl cis-trans isomerase	3	9	18,2	?
B8MWJ5	Probable alpha-galactosidase A	4	8	58,7	Yes
B8NIL7	ADP-ribosylation factor, putative	4	8	21,0	No
B8N9R6	Pyruvate carboxylase	4	8	131,1	?
P41747	Alcohol dehydrogenase 1	3	8	37,0	No
B8NSP9	Ribosomal protein L16a	3	8	23,0	No
B8NQJ3	Vacuolar aspartyl aminopeptidase Lap4, putative	3	8	55,5	?
B8NU00	NmrA-like family protein	3	8	36,7	?
B8NMV9	60S ribosomal protein L27	3	8	15,6	No
B8MYK9	IgE-binding protein	3	8	20,0	?
B8N8I7	Heterogeneous nuclear ribonucleoprotein G, putative	2	8	13,1	No
B8N5E0	Metalloprotease MEP1	2	8	25,2	?
B8N2I2	CFEM domain protein, putative	2	8	18,9	Yes/No
B8N7F1	Ribosomal protein S23 (S12)	2	8	15,8	No
B8NCT6	60S ribosomal protein L7	2	8	29,0	No
B8NTE9	Glycosyl hydrolases family 32 superfamily	2	8	67,1	?

B8MY51	60S ribosomal protein L22, putative	2	8	14,1	No
B8MWE7	60S ribosomal protein L9, putative	2	8	21,9	No
B8N023	60S ribosomal protein L35Ae	2	8	12,4	No
B8N9R3	60S ribosomal protein L11	2	8	20,0	No
B8NCW4	60S ribosomal protein L17	2	8	26,2	No
B8N5N1	Acid phosphatase, putative	2	8	33,0	?
B8NLM9	14-3-3 family protein artA	3	6	26,9	?
B8NFJ1	Peptide hydrolase	3	6	40,0	?
B8MZ41	Probable alpha/beta-glucosidase agdC	2	6	98,7	Yes
B8N439	RNA binding protein, putative	2	6	12,7	?
B8NW18	UDP-glucose 4-epimerase	2	6	25,6	?
B8N3U2	Glutaminase, putative	2	6	92,4	?
B8NNY3	40S ribosomal protein S17, putative	2	6	16,4	No
B8NGN0	Cytochrome c	2	6	12,1	No
B8NQL8	Ubiquinol-cytochrome C reductase complex core protein 2, putative	2	6	32,9	?
B8MYW2	TCTP family protein	2	6	19,7	?
B8NM07	Triosephosphate isomerase	2	6	27,4	?
B8MX84	Malate dehydrogenase, NAD-dependent	2	6	44,5	No
B8N2H3	Enolase/allergen Asp F22	2	6	47,4	No
B8NIR6	Pyridoxal reductase (AKR8), putative	2	6	34,6	?
B8NXN6	Alpha-1,2-mannosidase, putative subfamily	2	6	77,0	?
B8MVM1	Dyp-type peroxidase family protein	2	6	55,7	?
B8NFZ6	Endo-arabinase, putative	2	6	35,1	?
B8N3M3	Ribosomal protein L15	2	6	33,6	No
B8NKY3	Immunoglobulin G-binding protein H, putative	2	6	44,2	?
B8MZ48	Glutathione reductase	2	6	60,9	No
B8NIF5	D-lactate dehydrogenase, putative	2	6	51,4	?
B8N370	40S ribosomal protein S6	2	6	27,2	No
B8MW63	Gamma-glutamyltranspeptidase	2	6	62,9	?
B8N6U8	Mitochondrial processing peptidase beta subunit, putative	2	6	53,1	?
B8NLK5	Serine carboxypeptidase, putative	2	6	59,5	?
B8N521	Aldose 1-epimerase, putative	2	6	44,2	?
B8N5B0	Molecular chaperone Hsp70	2	6	69,7	?
B8N394	Acetyl-coenzyme A synthetase	3	5	78,9	?
B8NPM5	Laccase TilA	2	5	66,1	?
B8NIQ5	60S ribosomal protein L6	2	5	21,9	No
B8NUR2	60S ribosomal protein L21, putative	2	5	18,0	No
B8NA18	Cytochrome b-c1 complex subunit 7	2	5	14,3	No
B8NB42	40S ribosomal protein S11	2	5	15,8	No
B8NDH3	Fumarylacetoacetate hydrolase, putative	2	4	116,0	?
B8N4I0	6-phosphogluconate dehydrogenase, decarboxylating	2	4	54,2	?
B8NS47	Cell wall glucanase (Scw4), putative	2	4	35,9	?
B8NLP8	Alkaline phosphatase	2	4	66,6	No
B8MZV8	Regulatory protein SUAPRGA1	2	4	34,8	No
B8N9C3	Peroxidase	2	4	40,0	?
B8NA30	Allergen Asp F7	2	3	28,8	?
B8NCX4	Fasciclin domain family protein	2	3	53,1	?
B8NHG5	Allergenic ceratoplatanin Asp F13	2	3	22,8	Yes

B8NR98	60S ribosomal protein L4, putative	2	3	40,2	No
B8N4T2	Transketolase	2	3	74,8	?
B8MWA0	Pyruvate kinase	2	3	58,0	?

*The cellular localization of the proteins was referred to by the gene ontology (cellular component) entries on UniProt
Yes: The proteins are annotated to be on the conidial surface
No: The proteins are annotated at non-cell surface location
? No information available
PSMs: Peptide spectrum matches

Supplementary Table 2: The list of clinical isolates of *Aspergillus fumigatus* and *Aspergillus flavus* used in this study

Aspergillus fumigatus			***Aspergillus flavus***		
Number	Isolation		Number	Isolation	
	Year	Site		Year	Site
CBS144-89	(1)*	Lung	CI1698	2011	Eye
CNRMA13.623	2013	Eye	CNRMA8.318	2008	Eye
CNRMA18.547	2018	Eye	CNRMA13.781	2013	Eye
CNRMA16.511	2016	Eye	CNRMA8.1117	2008	Eye
CNRMA17.815	2017	Eye	CNRMA13.197	2013	Eye
CNRMA13.788	2013	Sinus	CNRMA6.994	2006	Sinus
CNRMA18.163	2018	Sinus	CNRMA15.797	2015	Sinus
CNRMA15.022	2014	Sinus	CNRMA13.195	2013	Sinus
CNRMA17.623	2017	Sinus	CNRMA15.021	2014	Sinus
CNRMA13.349	2013	Lung	CNRMA18.189	2018	Lung
CNRMA15.354	2015	Lung	CNRMA18.659	2018	Lung
CNRMA14.564	2014	Lung	CNRMA18.541	2018	Lung
CNRMA15.011	2014	Lung	CNRMA18.657	2018	Lung

(1)* Monod, M., Togni, G., Rahalison, L., and Frenk, E. (1991) Isolation and characterisation of an extracellular alkaline protease of Aspergillus fumigatus. *J Med Microbiol* **35**, 23-28

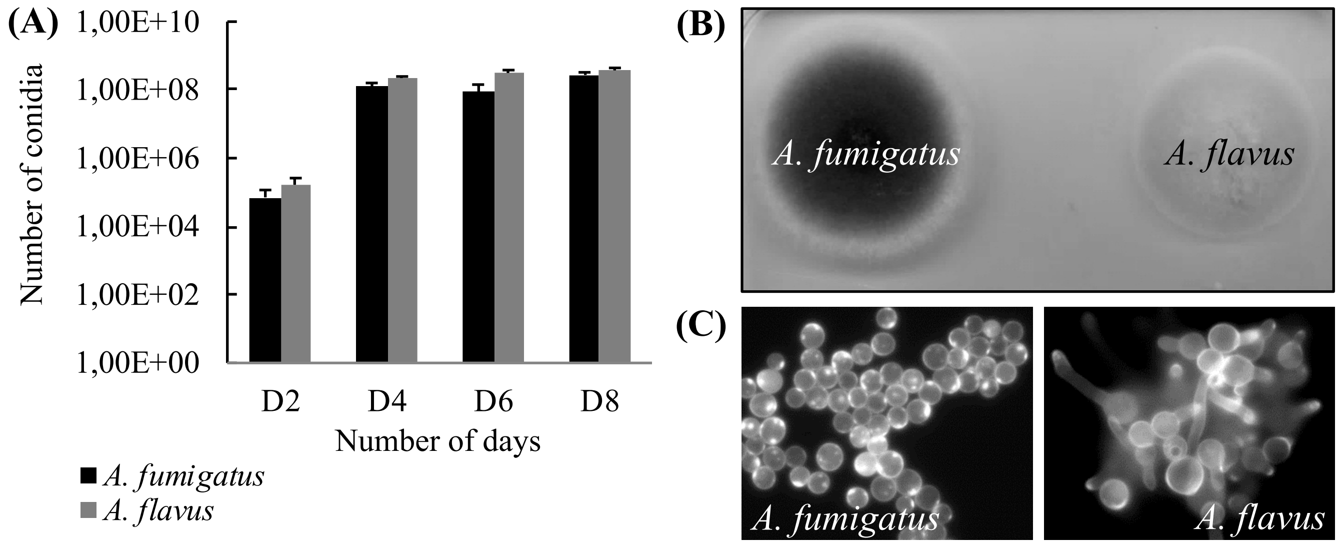

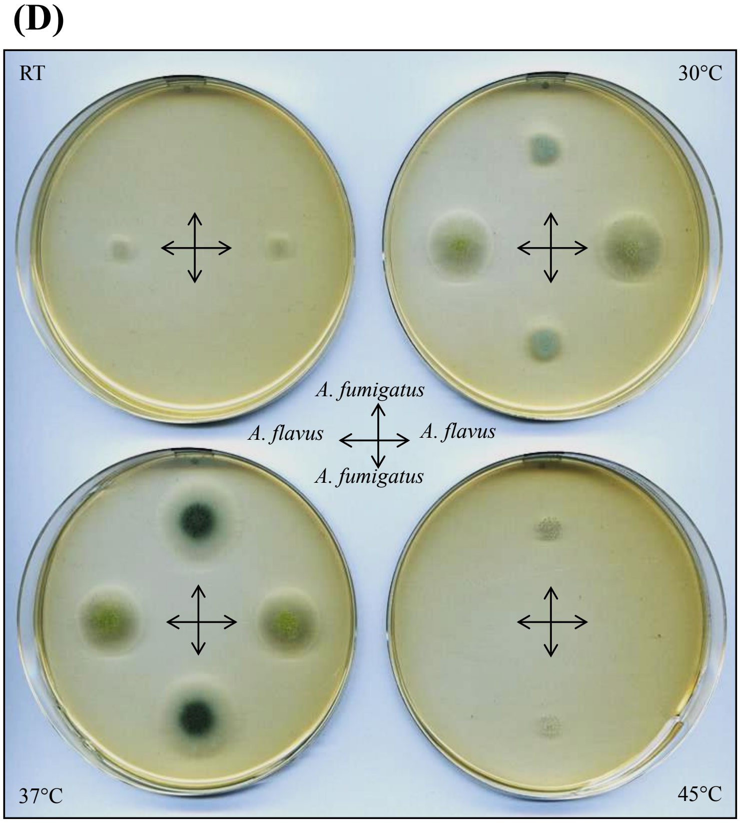

Supplementary Figure 1: Conidiation and germination of the two *Aspergillus* species. (A) There was no difference in conidiation between the two *Aspergillus* species. **(B)** The radial growth of *A. fumigatus* on Sabouraud agar medium was relatively faster than that of *A. flavus*. **(C)** In Sabouraud liquid medium, contrary to the Sabouraud-agar medium, the germination of *A. fumigatus* was relatively slower than that of *A. flavus* after 6 h at 37°C. Cells were labeled by Calcofluor White. **(D) Fungal growth on solid agar medium at different temperature.** *A. flavus* could grow on malt-agar medium at lower temperature (ambient temperature to 37°C) but not at higher temperature tested (45°C). *A. fumigatus* showed better growth at higher temperatures tested (30-45°C). At least two-three biological replicates were performed for all these experiments.

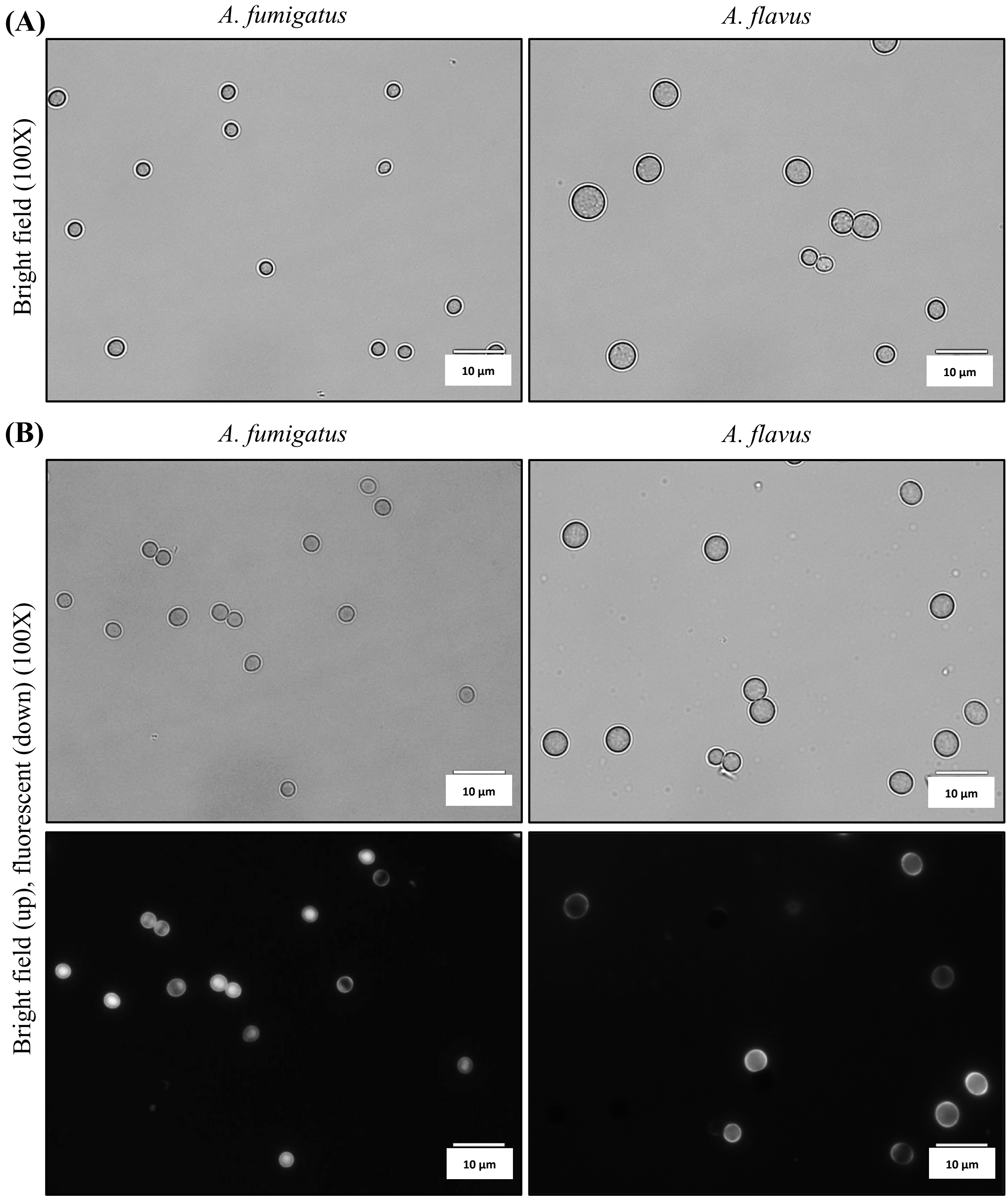

Supplementary Figure 2: Phenotypic differences between *A. fumigatus* and *A. flavus* conidia. (A) Bright field microscopy showed that the conidial sizes of the two *Aspergillus* species are different. *A. fumigatus* conidia and *A. flavus* conidia were 2.6 ± 0.2 µm and 5.2 ± 1.9 µm, respectively, in diameter. **(B)** *A. fumigatus* conidia were uniformly labelled with Calcofluor White (CFW), whereas *A. flavus* conidia showed heterogeneous labelling. At least three biological replicates were performed for these experiment, and each time a minimum of five images were captured for a sample.

Supplementary Figure 3: Conidial labeling with (A) Concanavalin A (ConA) and (B) Wheat Germ Agglutinin (WGA), both conjugated with fluorescein isothiocyanate (FITC). *A. fumigatus* conidia were negative for both ConA-FITC and WGA-FITC labeling, while *A. flavus* conidia showed heterogeneous labeling. At least two biological replicates were performed for labeling experiment, and each time a minimum of five images were captured for a sample.

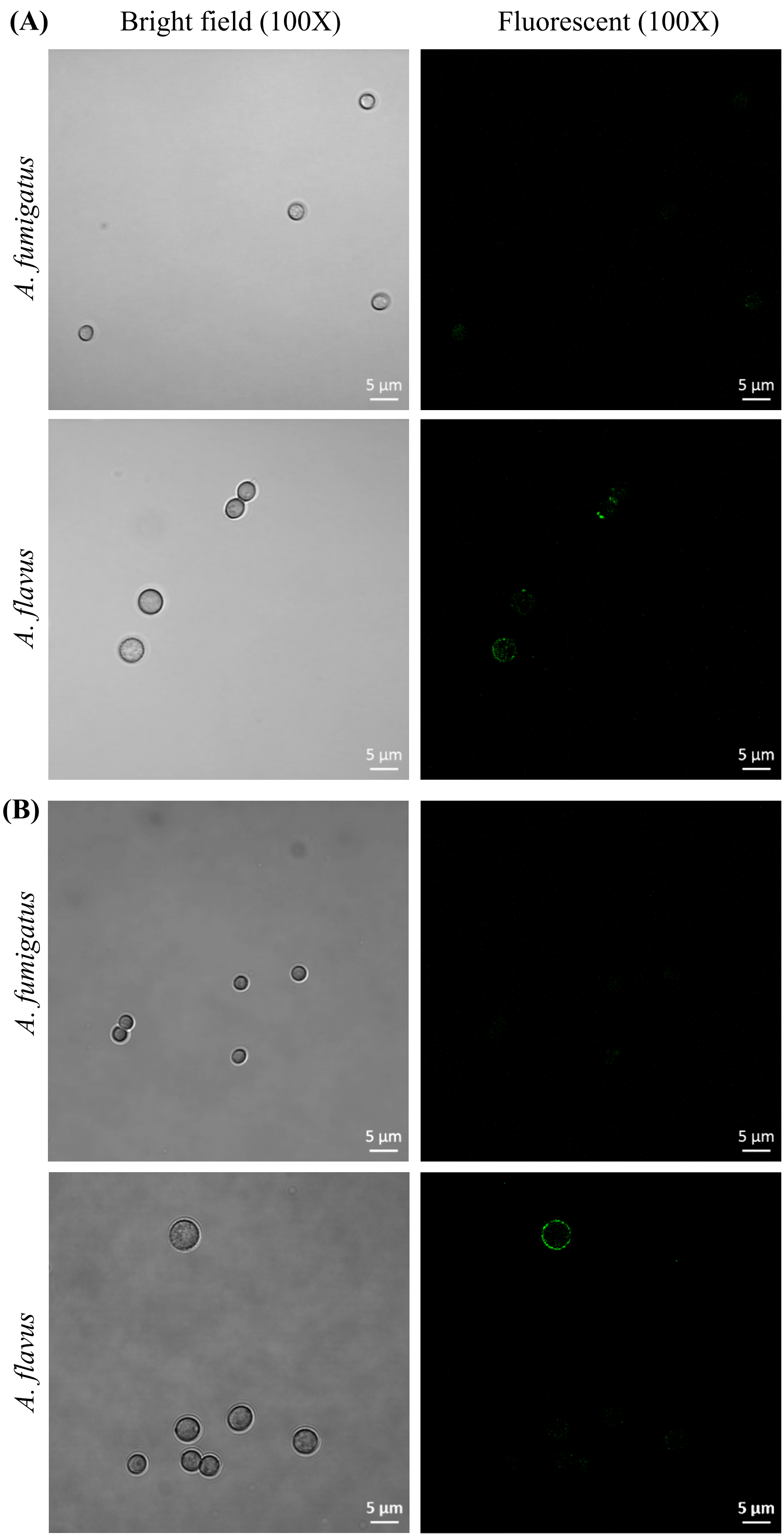

(A)

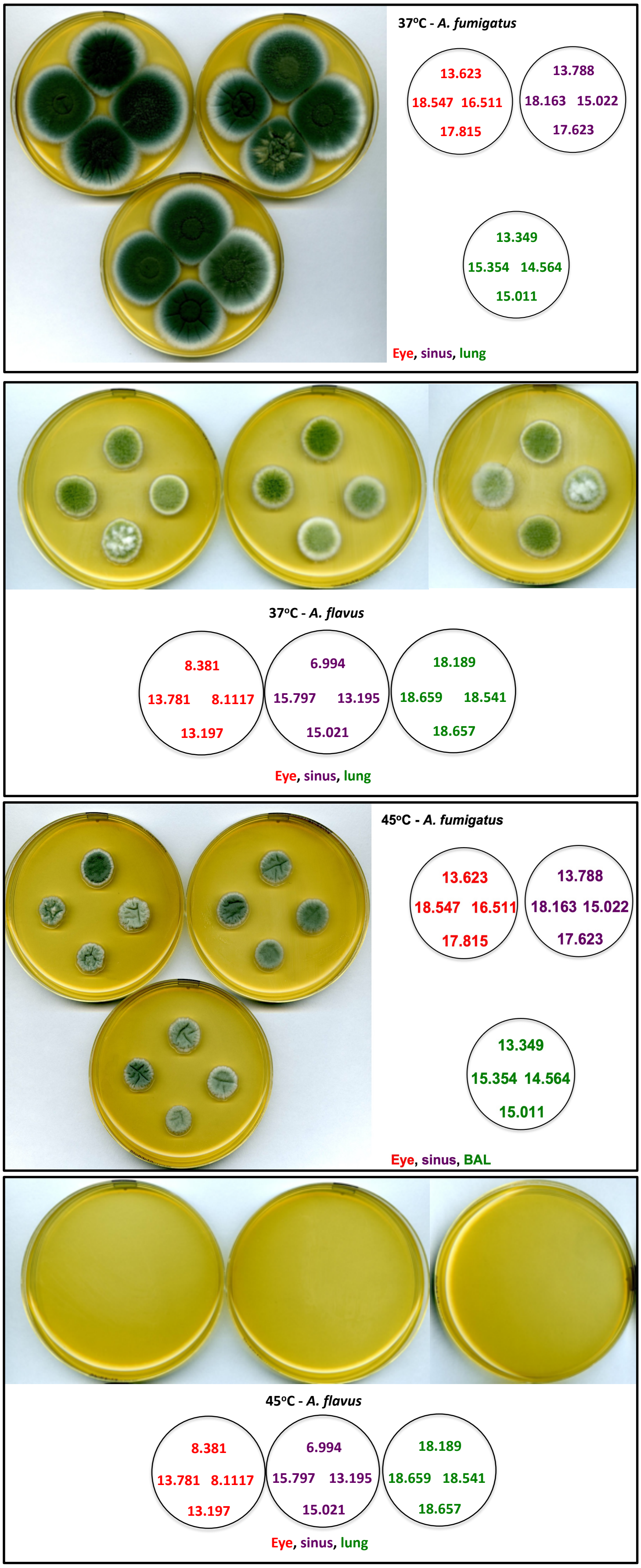

(B)

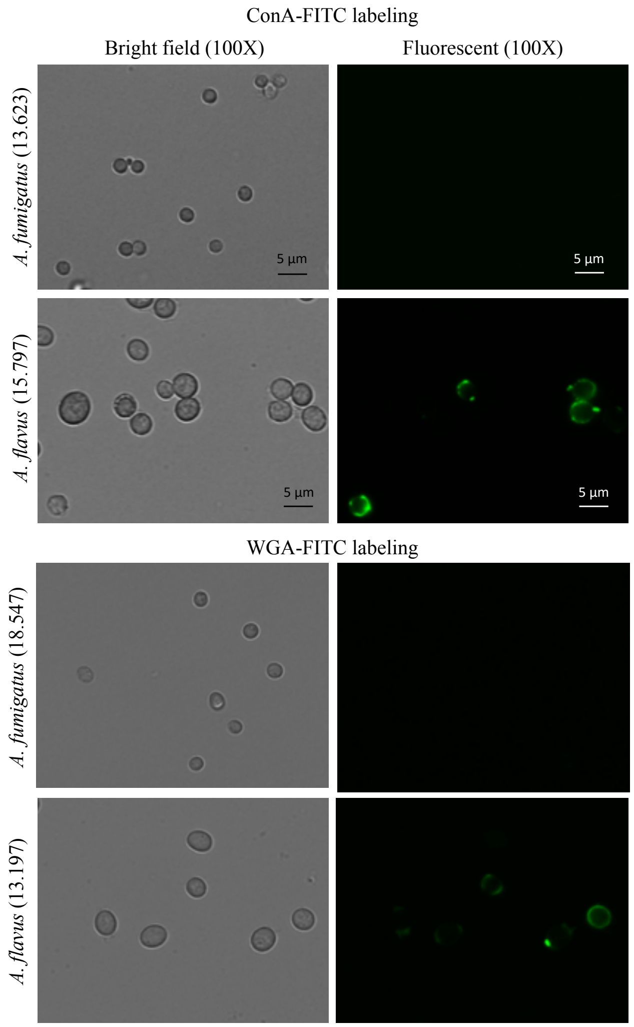

Supplementary Figure 4: Growth and immunolabeling of the different clinical isolates of *A. fumigatus* and *A. flavus.* (A) Twelve clinical isolates of *A. fumigatus* and *A. flavus* isolated from different sites of infection showed growth at different temperature similar to CBS144-89 and CI1698 strains of *A. fumigatus* and *A. flavus*, respectively. This was performed at least twice **(B)** Conidial surface labeling with ConA-FITC/WGA-FITC; one representative clinical isolates of *A. fumigatus* and *A. flavus* are presented. At least two biological replications were performed for labeling experiment, and each time a minimum of five images were captured for a sample.

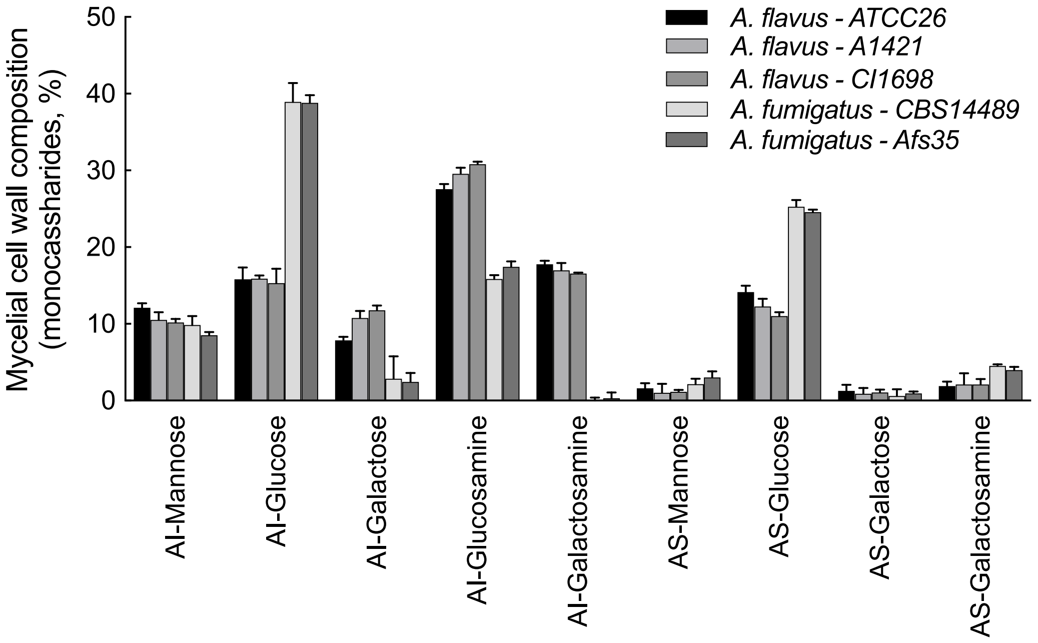

Supplementary Figure 5: Cell wall compositions of *A. fumigatus* (CBS-144-89 and Afs35) and *A. flavus* (ATCC26, A1421 and CI1698) strains. Mycelial cell wall composition of a clinical isolate of *A. fumigatus*, Afs35, was similar to that of CBS144-89 strain, whereas the ATCC strain and a clinical isolate of *A. flavus* were similar to that of *A. flavus* CI1698 strain (AI is the alkali-insoluble fraction and AS represents alkali-soluble fraction; Mannose and Galactose are the components of galactomannan; AI-Glucose and AS-Glucose represent β-1,3-glucan and α-1,3-glucan, respectively; chitin content is represented by AI-Glucosamine; AI/AS-Galactosamine and Galactose are the components of the galactosaminogalactan (GAG). Three independent experiments were performed to obtain these cell-wall monosaccharide compositions.

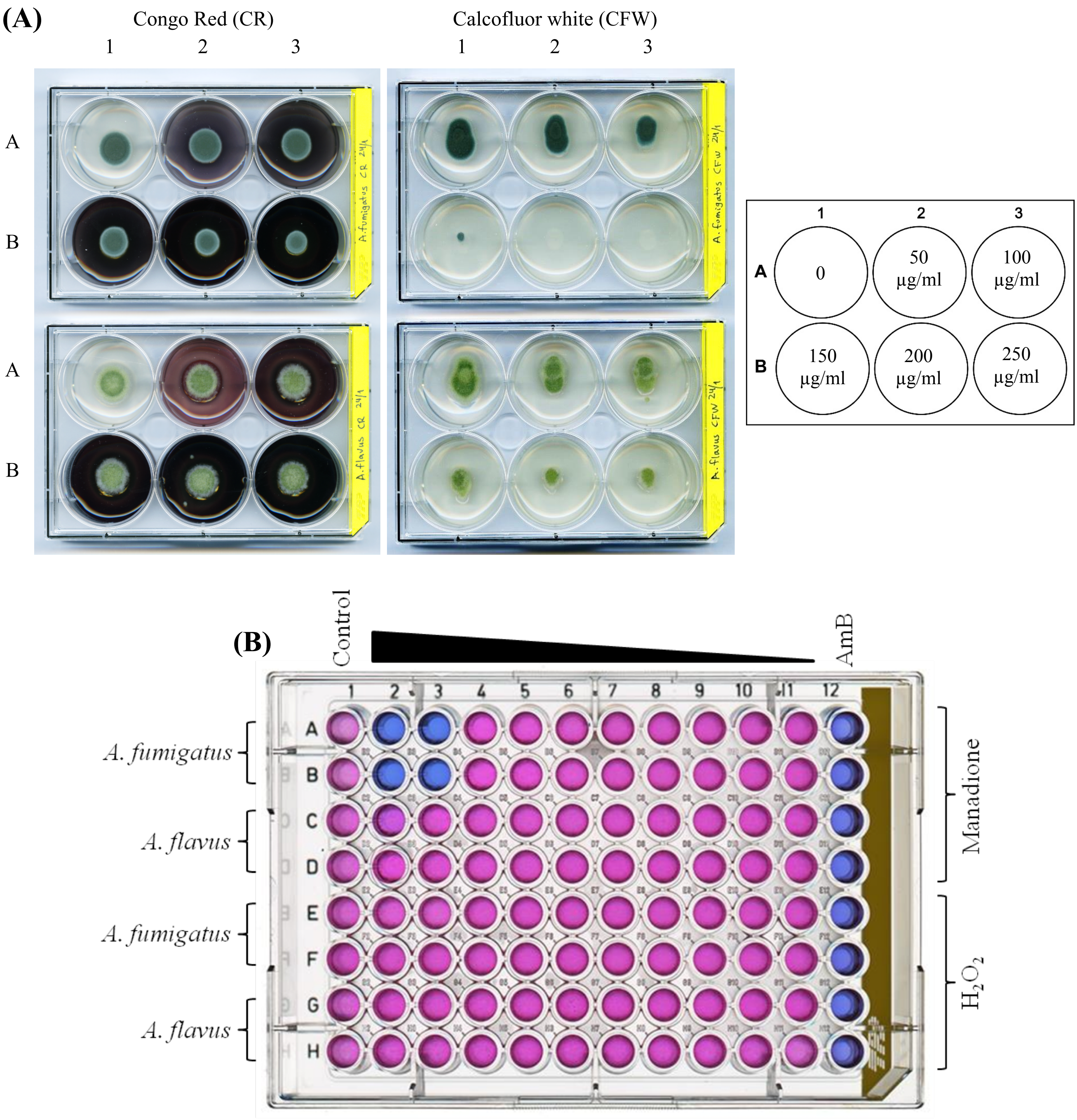

Supplementary Figure 6: (A) Susceptibility to cell wall perturbing agents. *A. flavus* was not sensitive to the cell wall perturbing agents tested (CFW and CR); while *A. fumigatus* was sensitive to higher concentrations of CFW (>150 µg/mL). **(B) Growth assay using resazurin in the presence of oxidative stress inducing agents.** *A. fumigatus* and *A. flavus* were grown in the presence of two-fold dilutions of menadione (highest concentration – 160 µM) or H_2O_2 (highest concentration – 6 µM) and their growths were assessed by resazurin method. Amphotericin B (AmB; 2 µg/well) was used as the positive control for growth inhibition. This experiment was repeated twice.
